# Supplementary figures and images for: Identification of potential biomarkers associated with immune cell infiltration patterns in Kawasaki disease via bioinformatics
Source: PLoS One. 2025 Jun 2;20(6):e0324337. doi: 10.1371/journal.pone.0324337 (PMC12129191; doi:10.1371/journal.pone.0324337)

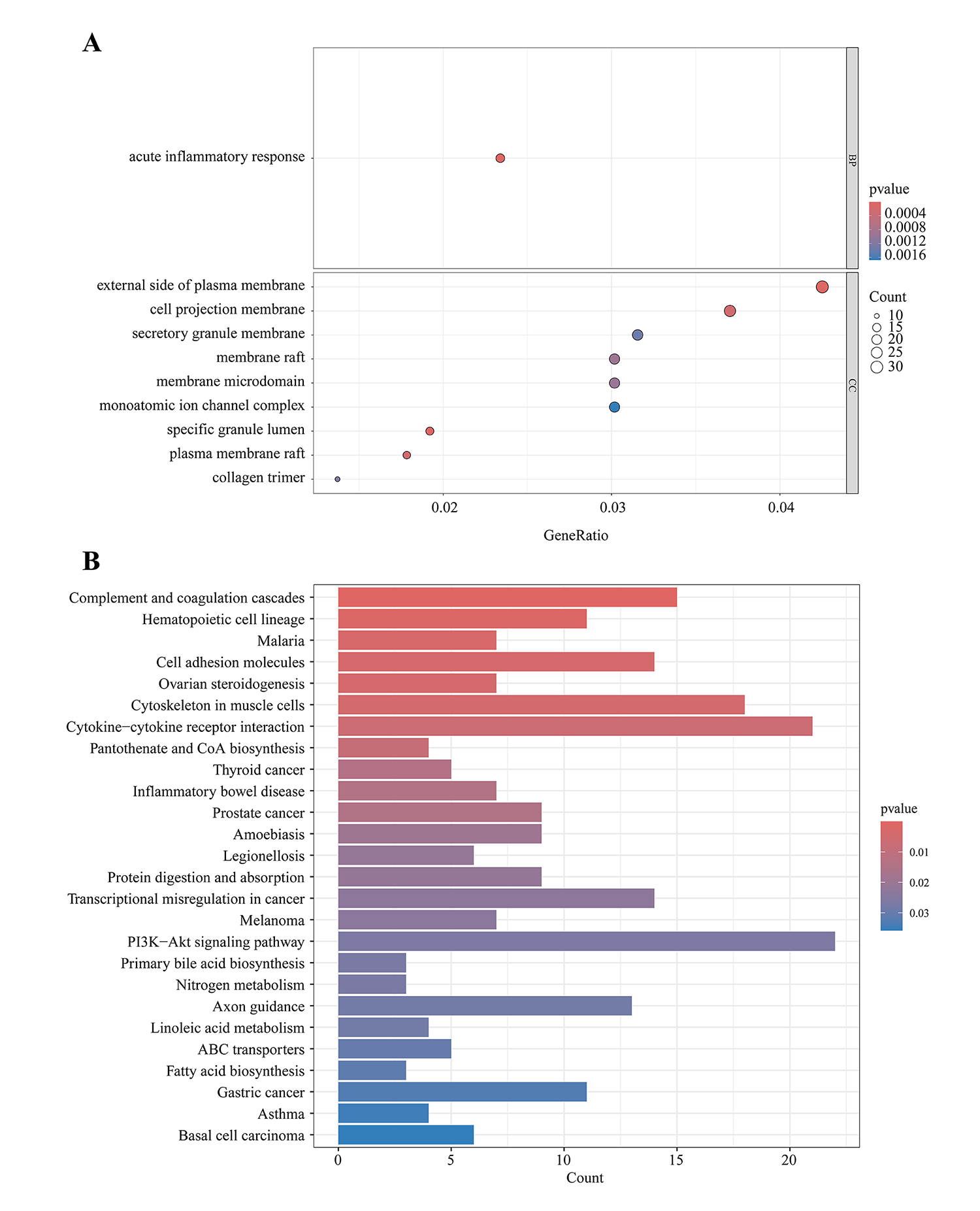

Supplement: S1 Fig — (TIF) [file pone.0324337.s001.tif]

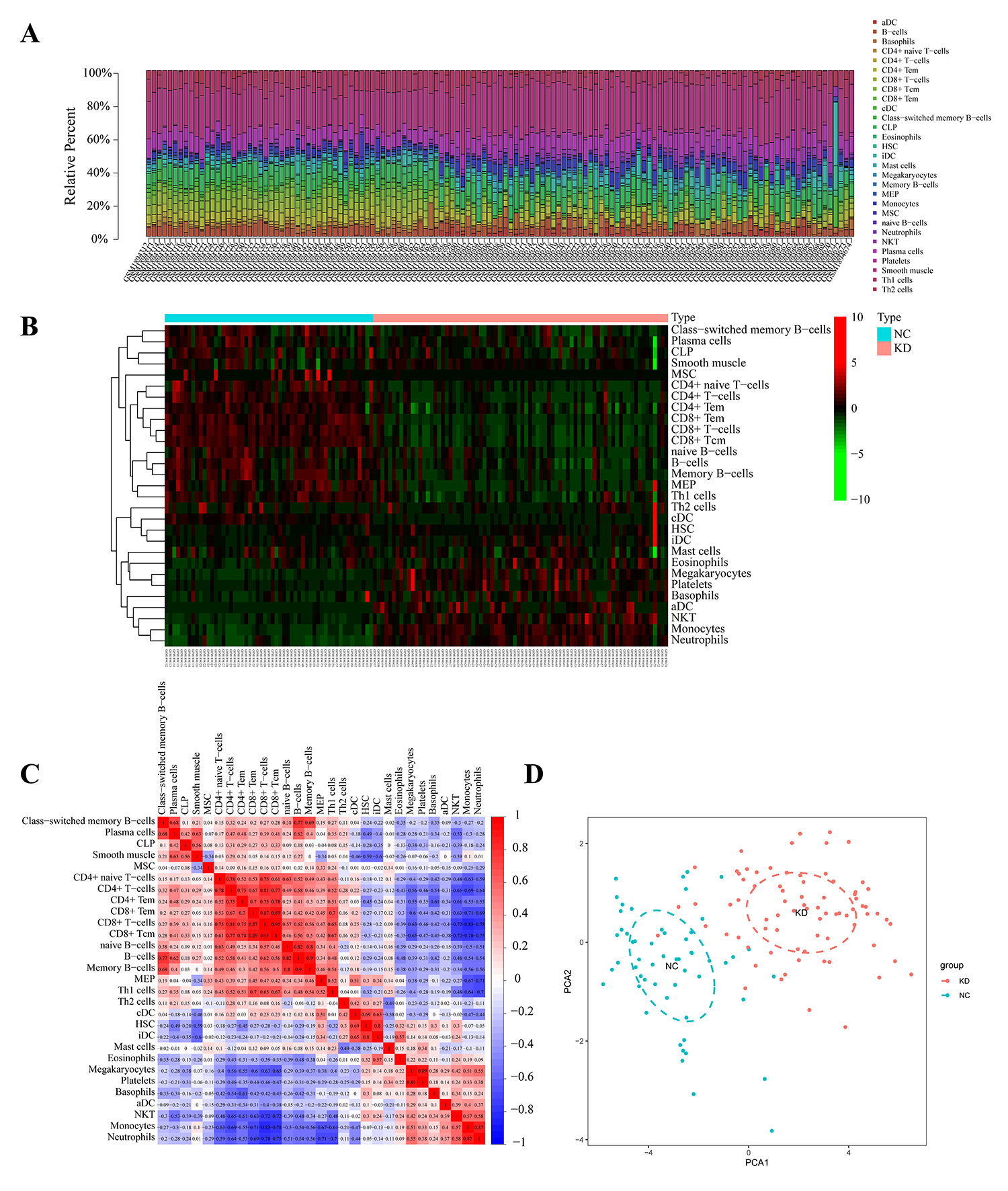

Supplement: S2 Fig — (TIF) [file pone.0324337.s002.tif]

Type  
对照组  
病例组

5

0

-5

Type

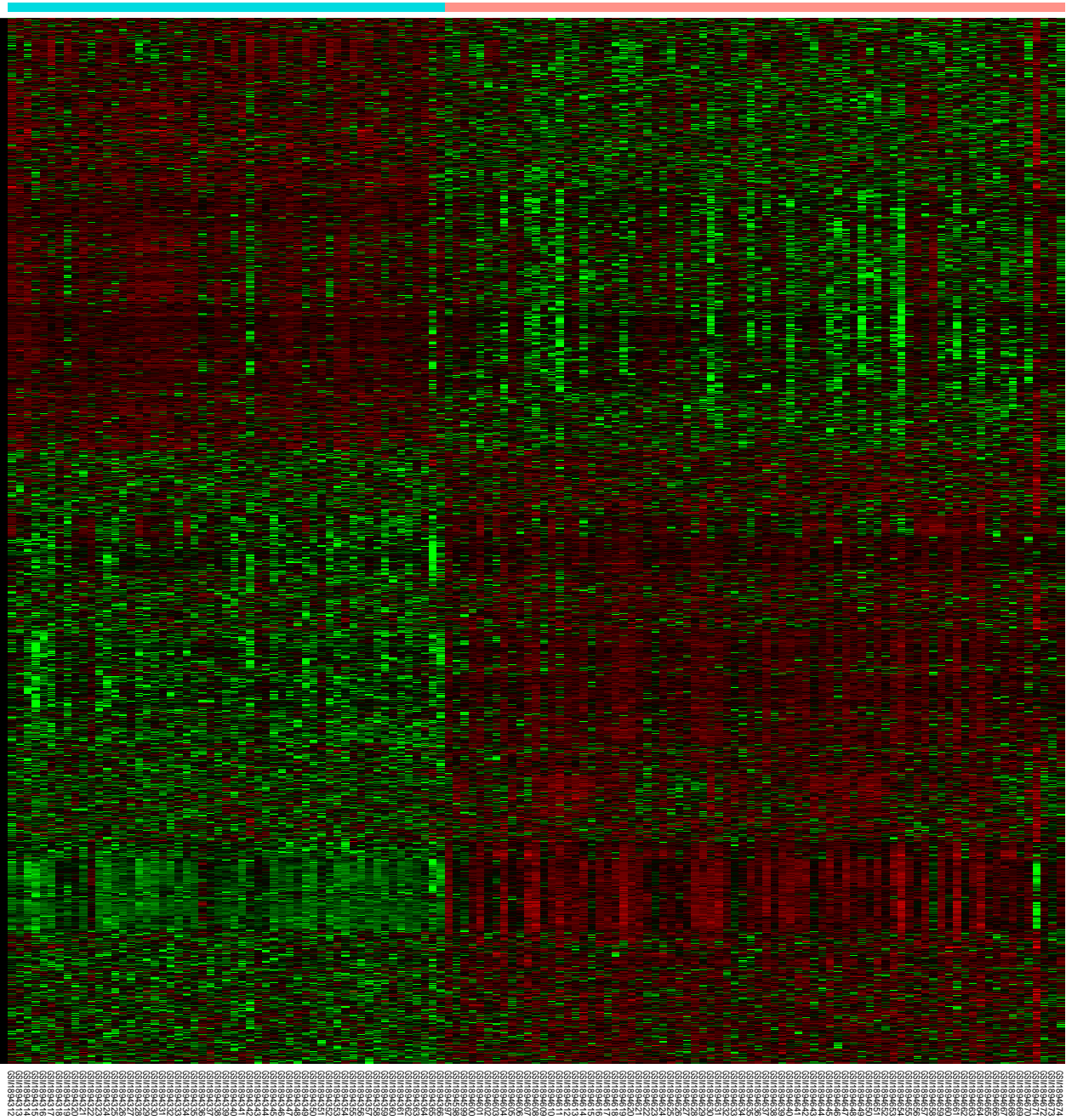

Supplement: S4 File — (ZIP) [file pone.0324337.s006.zip › 4. Differential Analysis/mrnaHeatmap.pdf]

# Volcano Plot

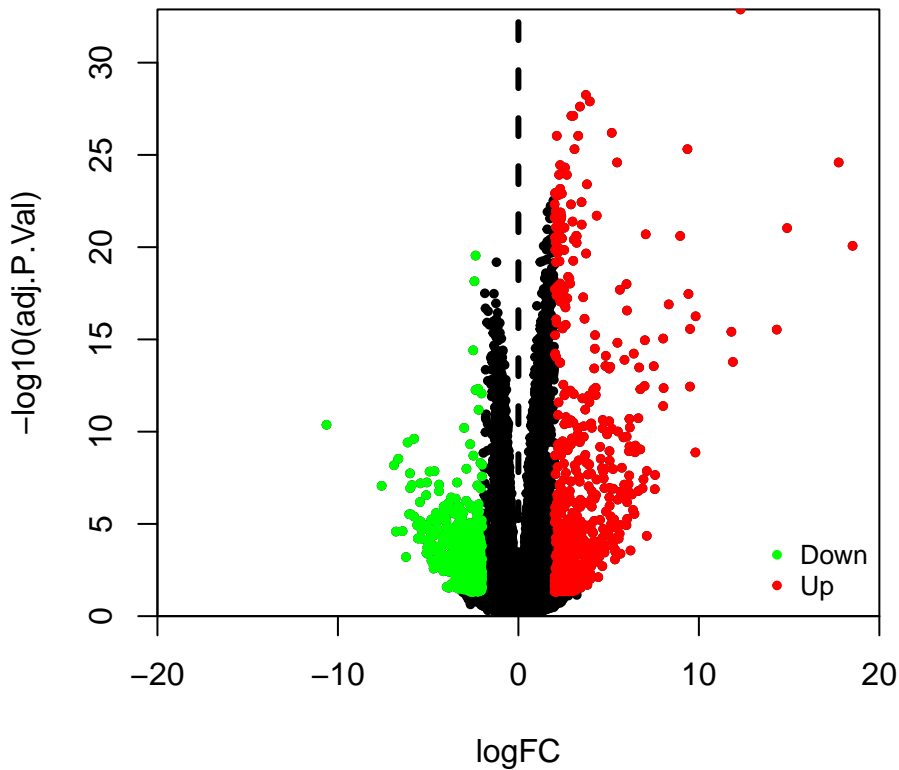

Supplement: S4 File — (ZIP) [file pone.0324337.s006.zip › 4. Differential Analysis/mrnaVol.pdf]

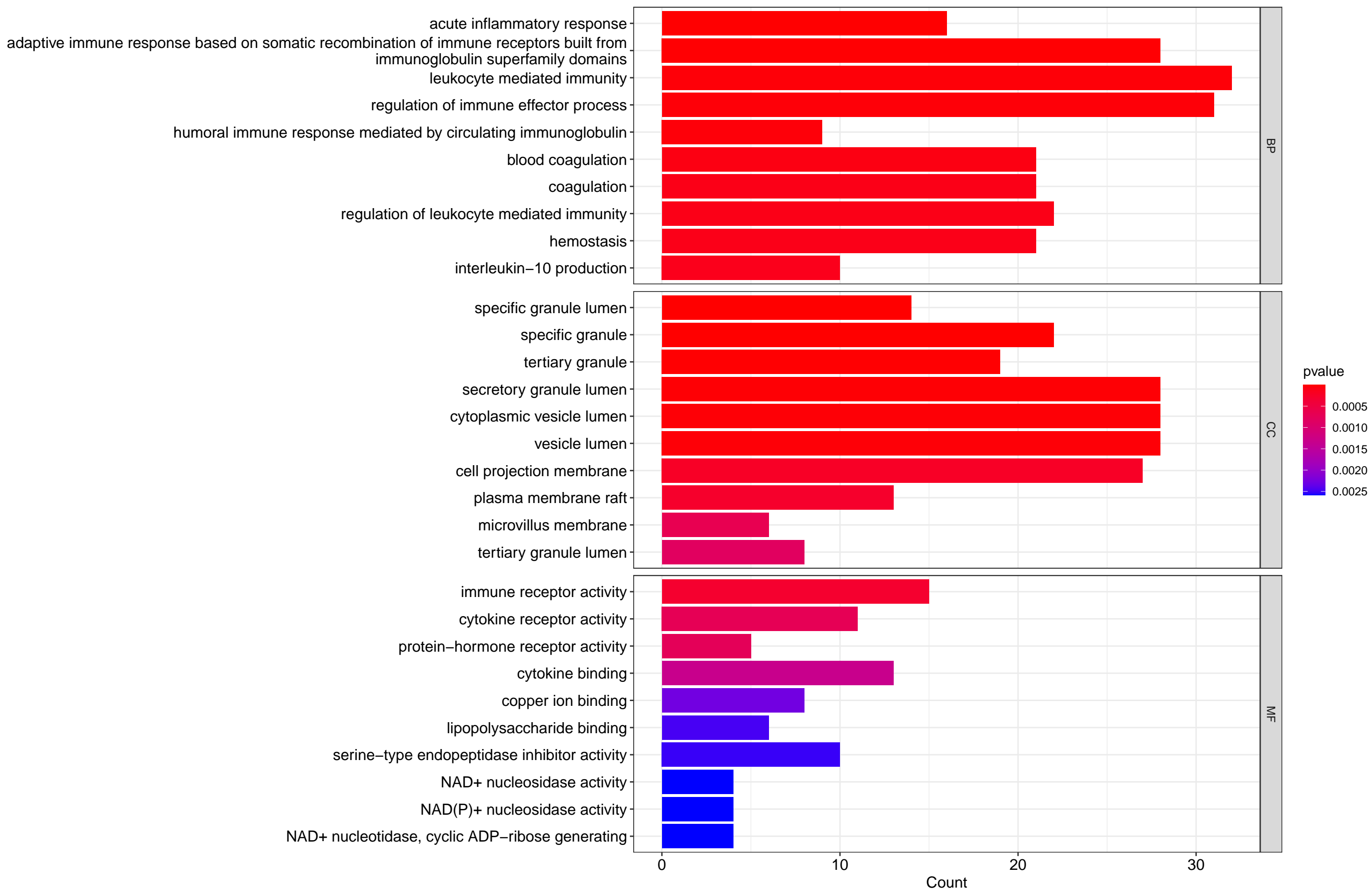

Supplement: S5 File — (ZIP) [file pone.0324337.s007.zip › 5. Gene Name to ID Conversion/barplot.pdf]

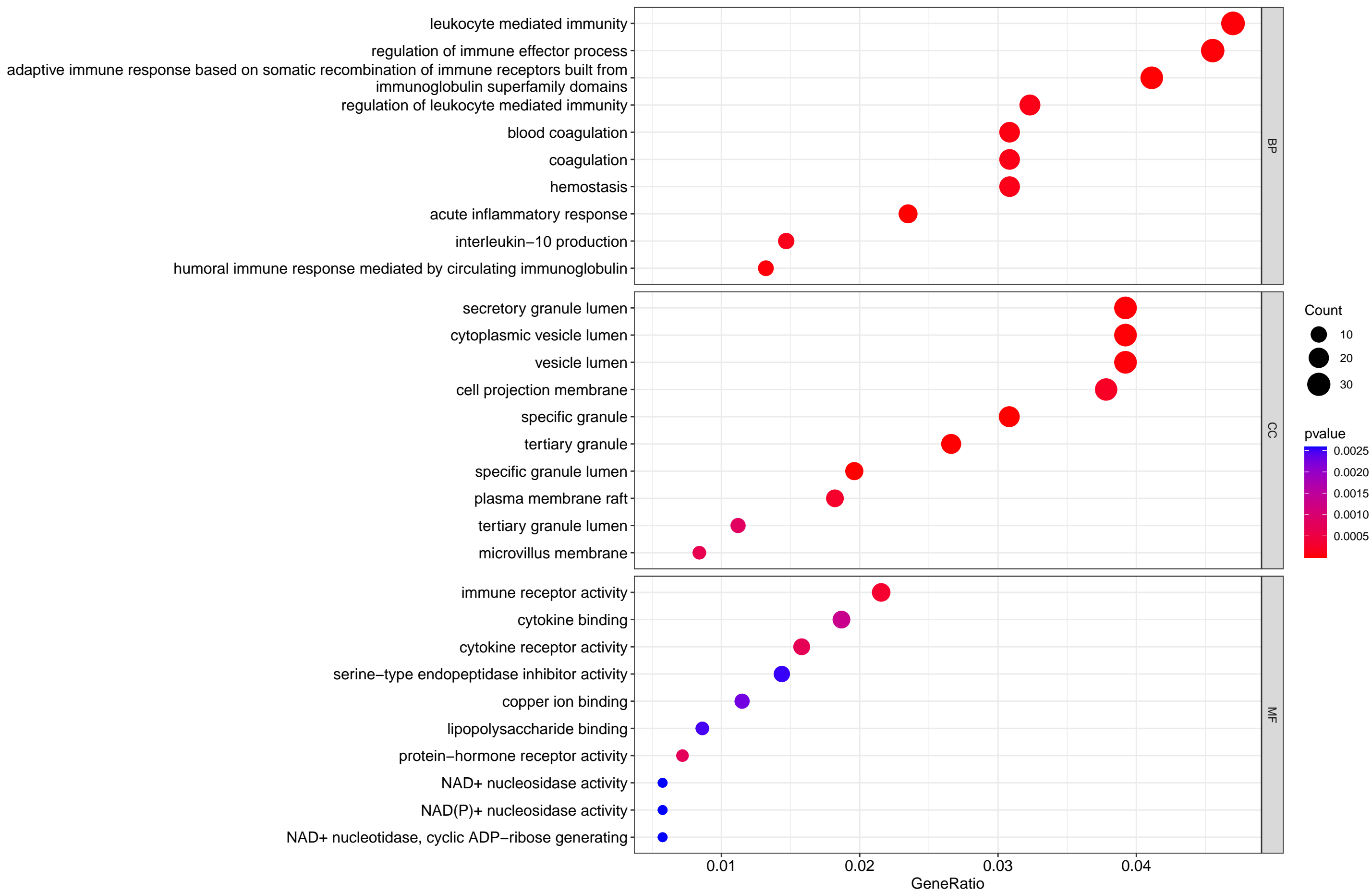

Supplement: S5 File — (ZIP) [file pone.0324337.s007.zip › 5. Gene Name to ID Conversion/bubble.pdf]

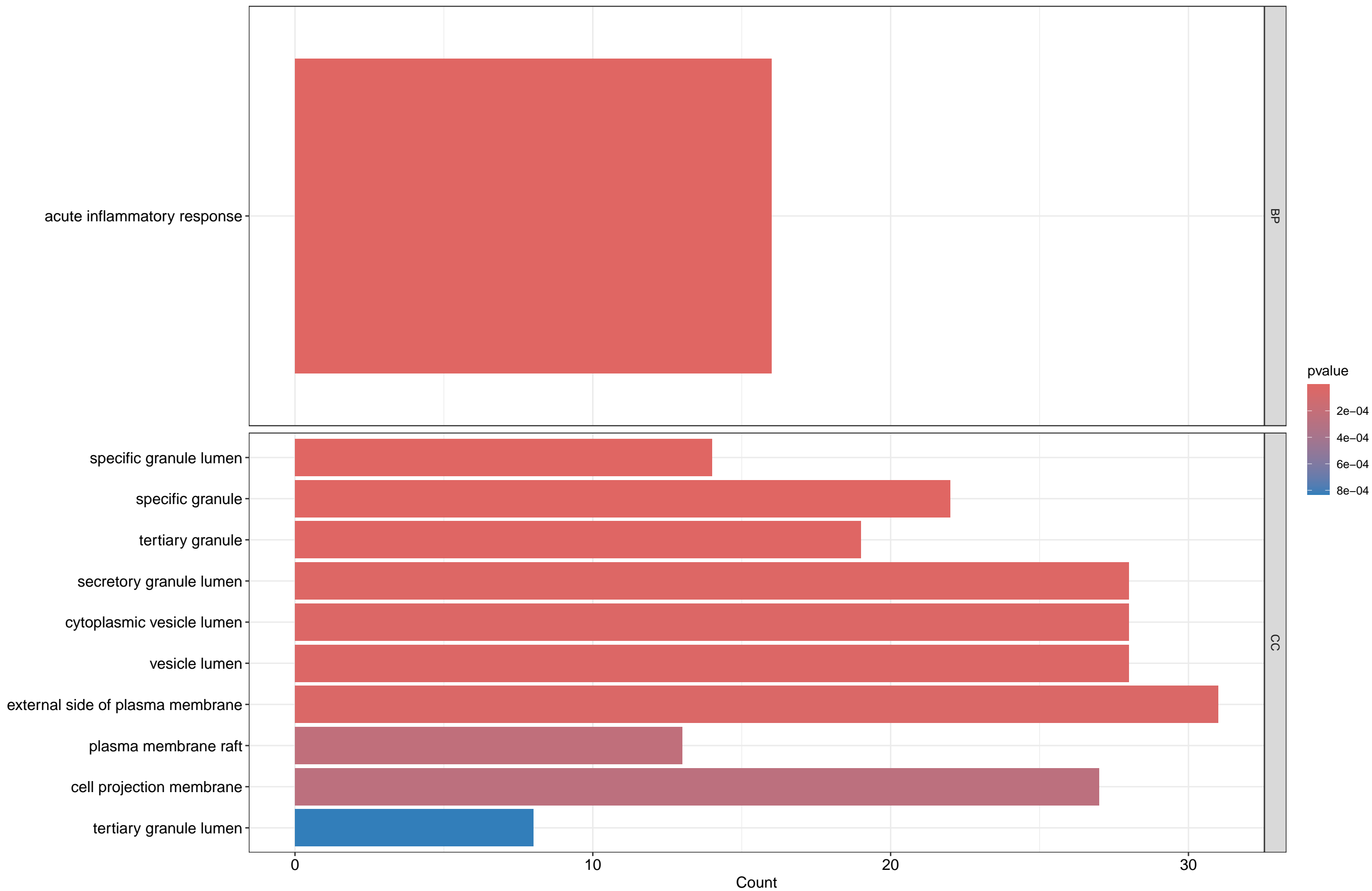

Supplement: S6 File — (ZIP) [file pone.0324337.s008.zip › 6. GO Enrichment Analysis/barplot.pdf]

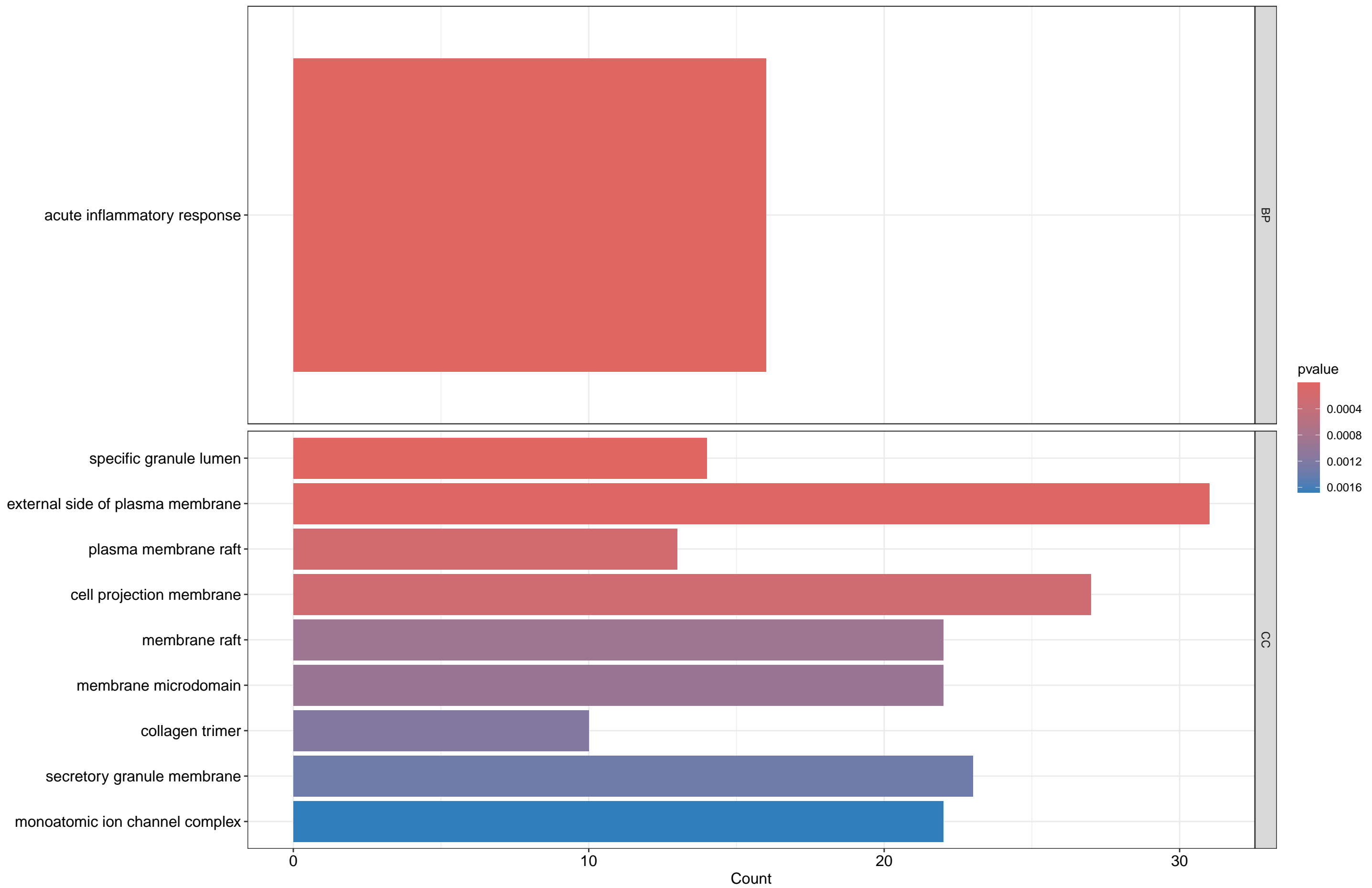

Supplement: S6 File — (ZIP) [file pone.0324337.s008.zip › 6. GO Enrichment Analysis/barplot_simplified.pdf]

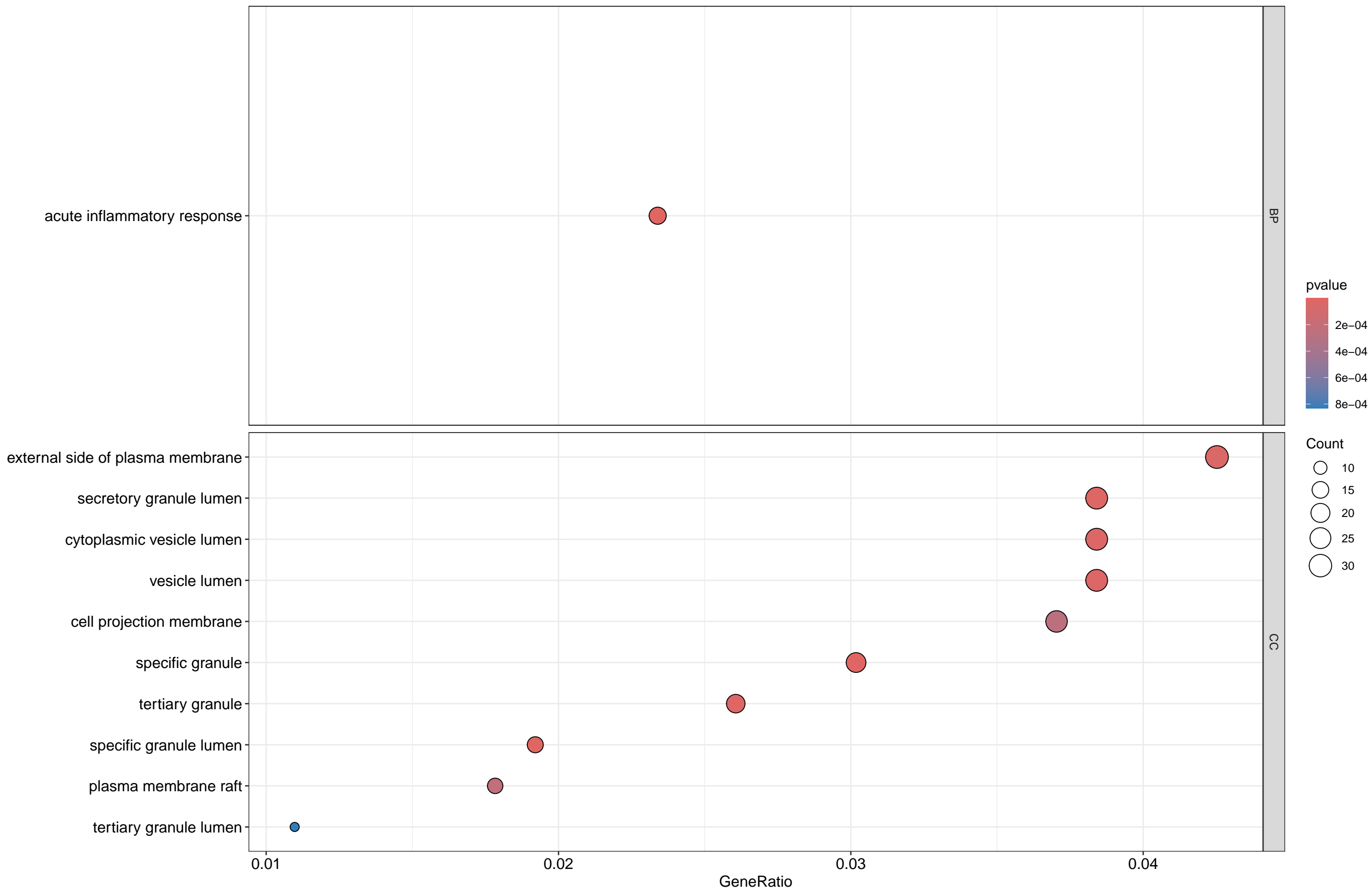

Supplement: S6 File — (ZIP) [file pone.0324337.s008.zip › 6. GO Enrichment Analysis/bubble.pdf]

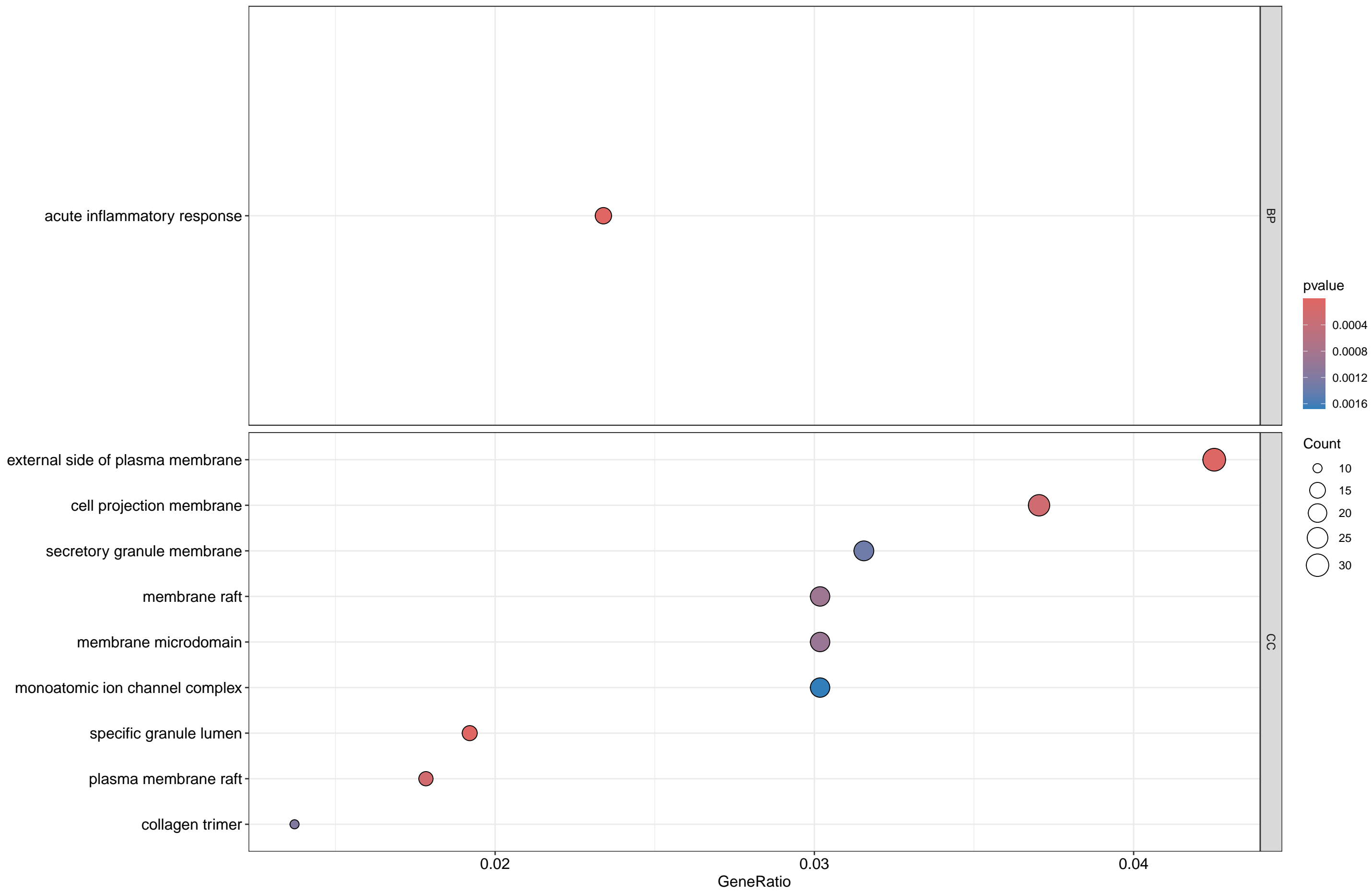

Supplement: S6 File — (ZIP) [file pone.0324337.s008.zip › 6. GO Enrichment Analysis/bubble_simplified.pdf]

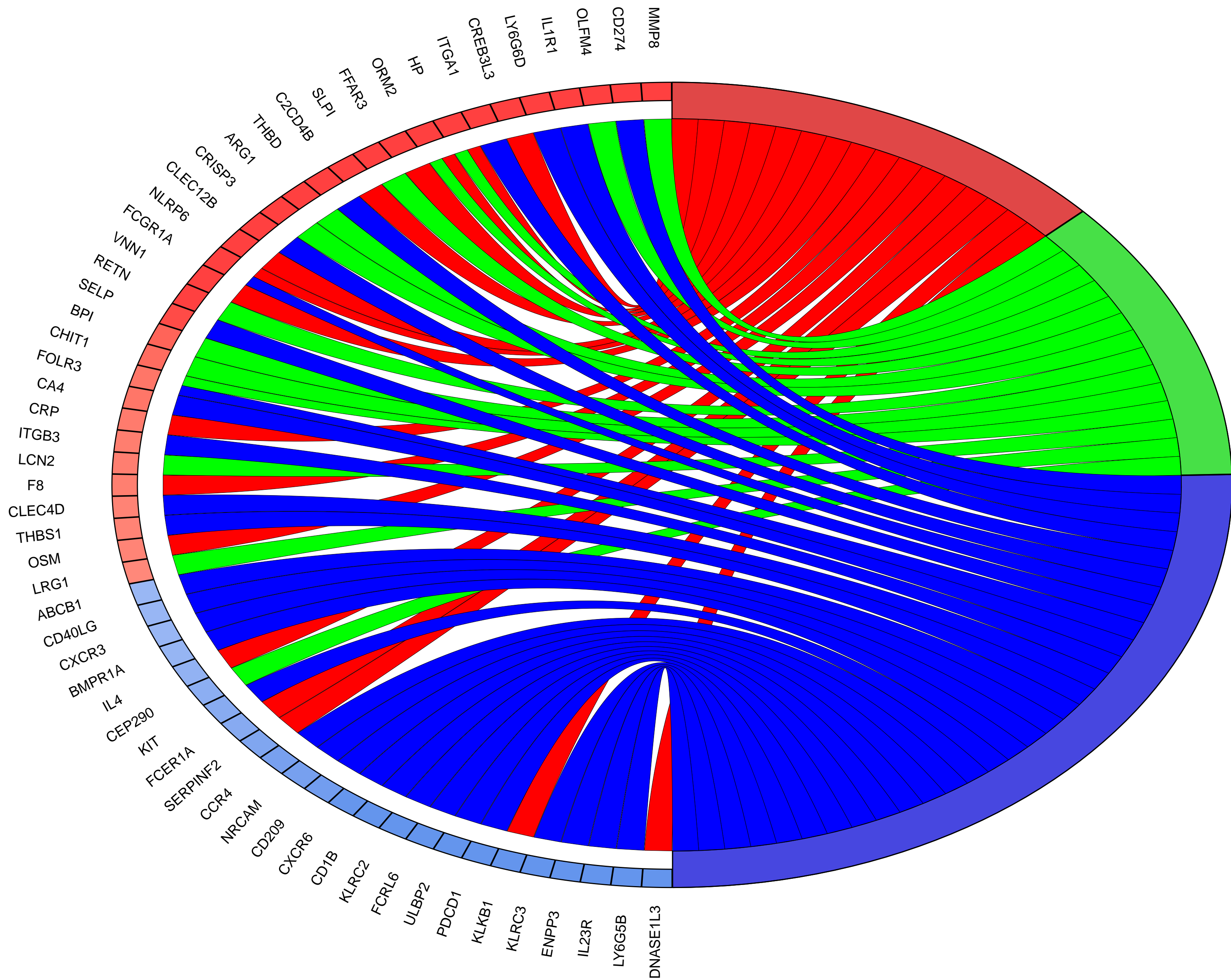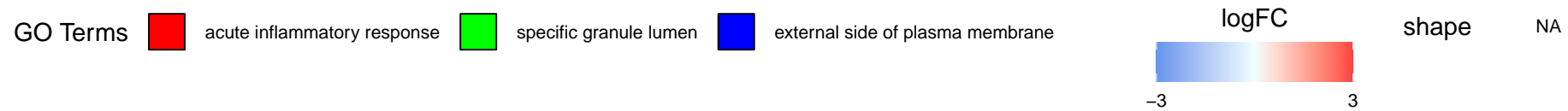

Supplement: S7 File — (ZIP) [file pone.0324337.s009.zip › 7. GO Diagram/circ.pdf]

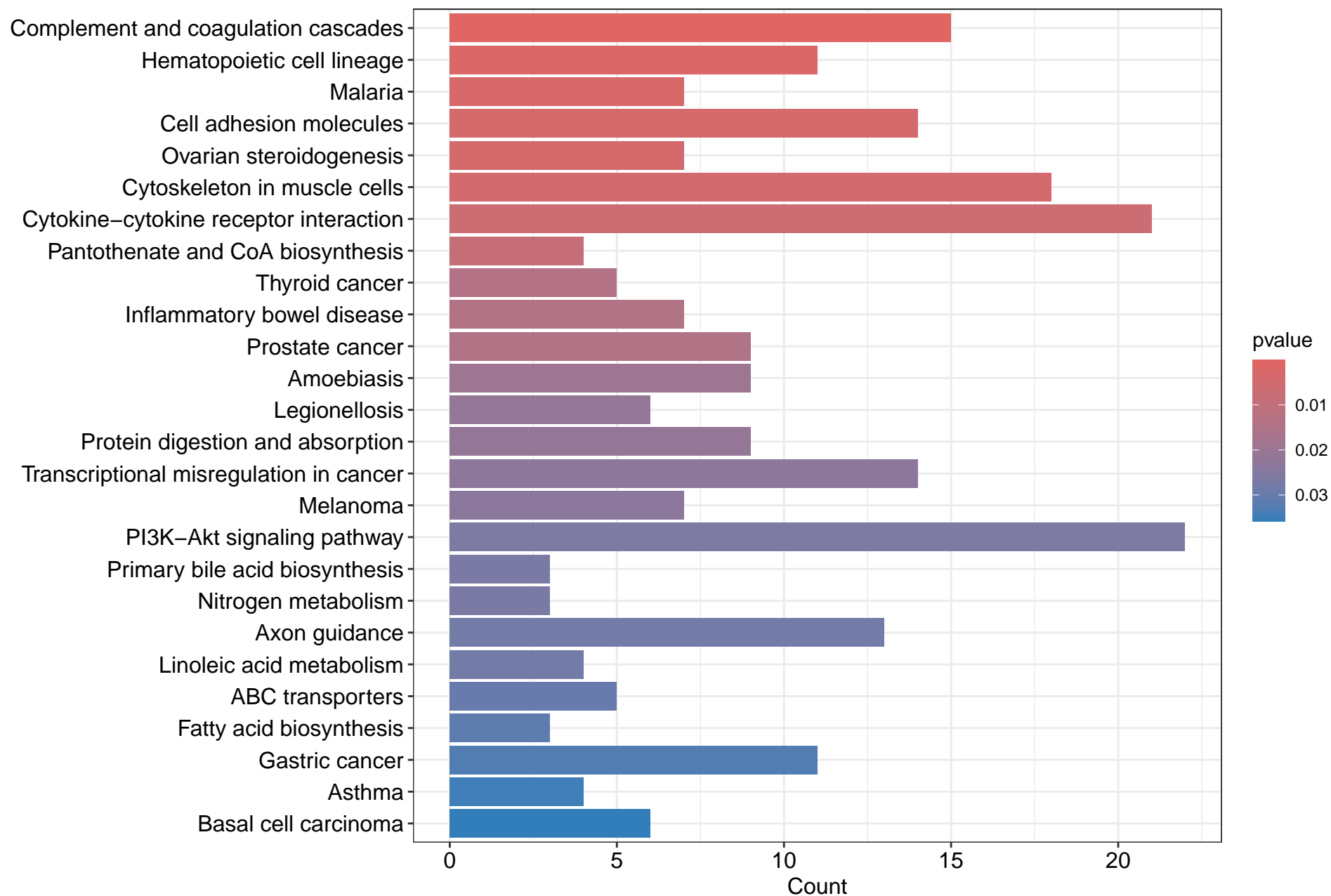

Supplement: S8 File — (ZIP) [file pone.0324337.s010.zip › 8. KEGG Enrichment Analysis/barplot_before.pdf]

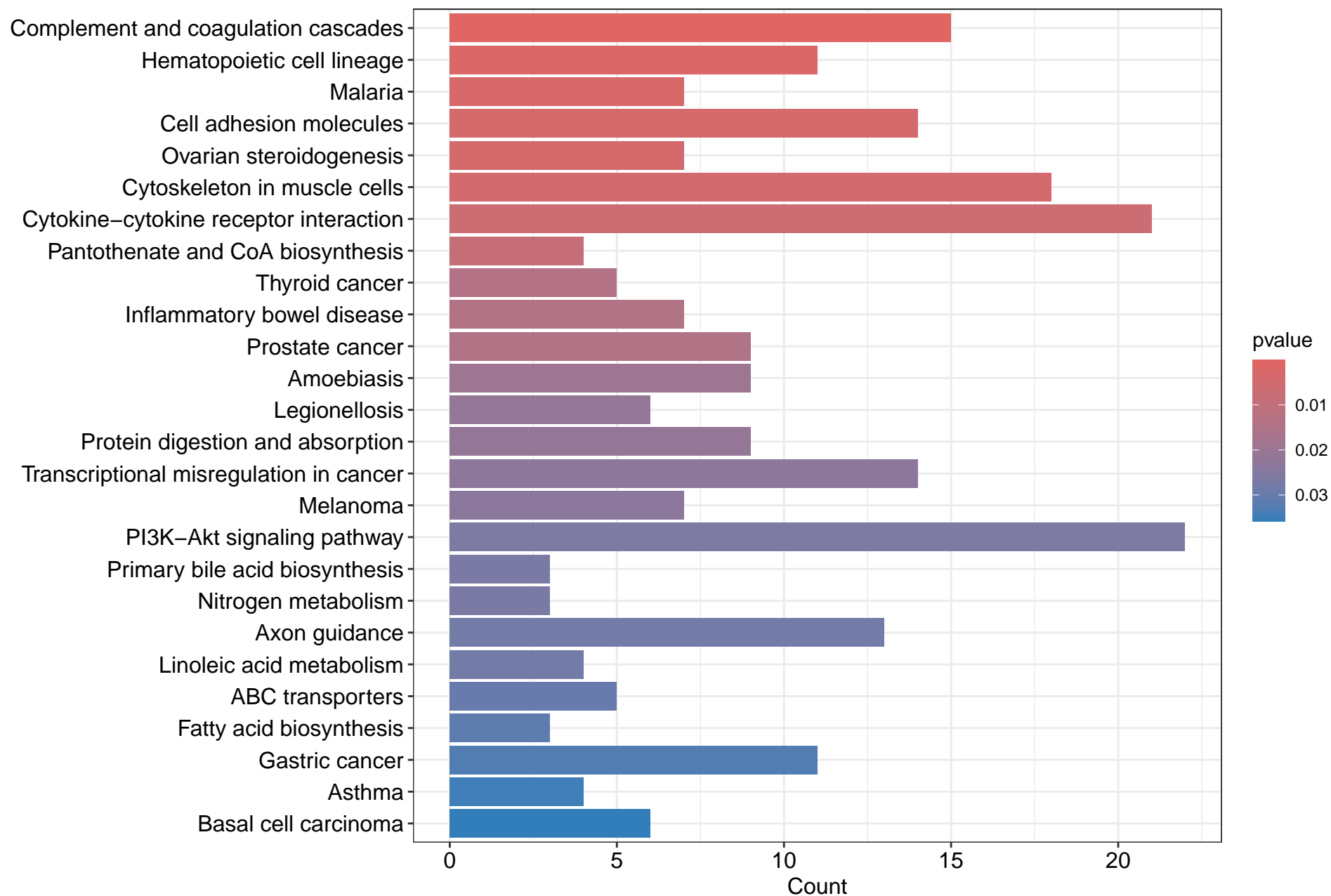

Supplement: S8 File — (ZIP) [file pone.0324337.s010.zip › 8. KEGG Enrichment Analysis/barplot_simplified.pdf]

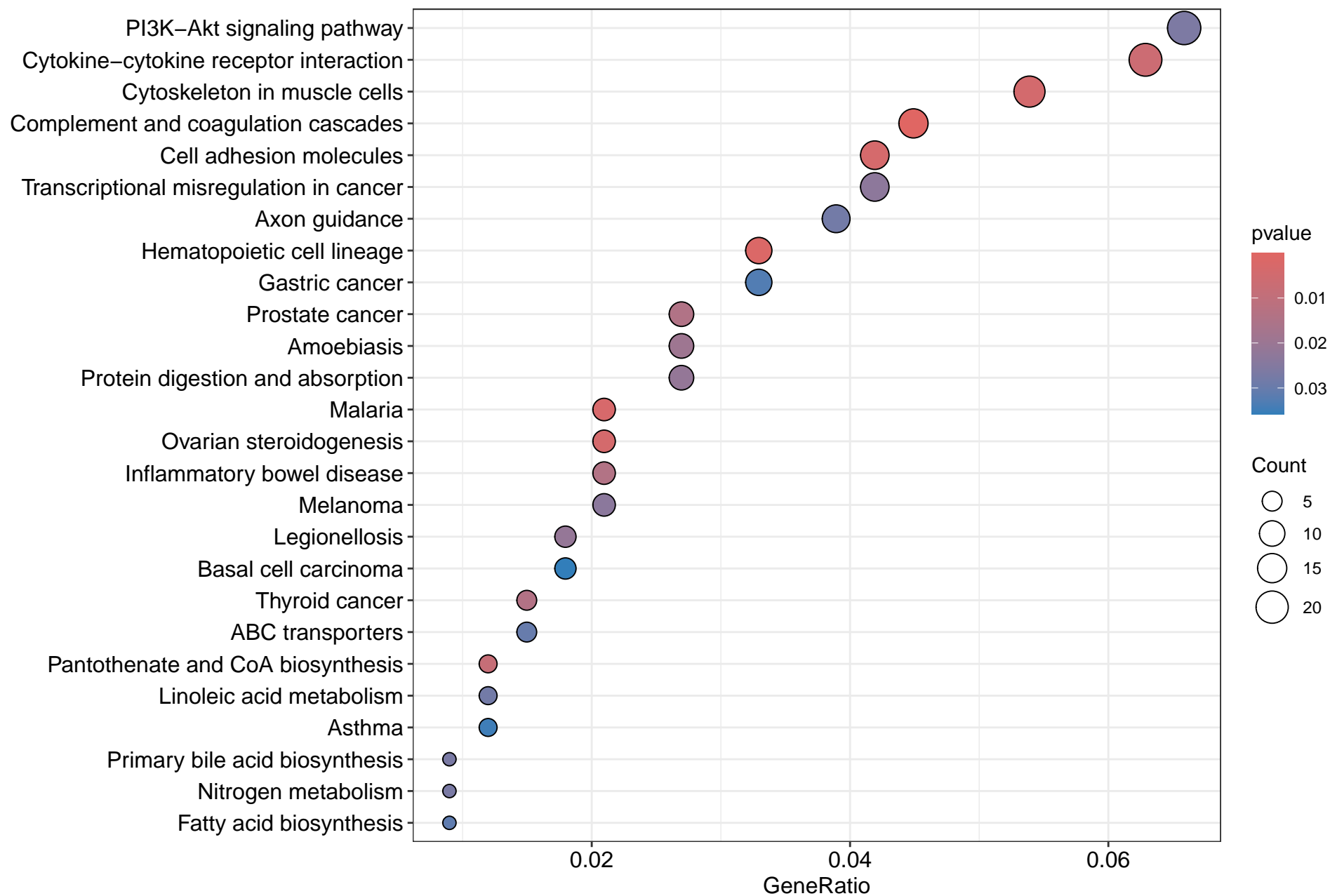

Supplement: S8 File — (ZIP) [file pone.0324337.s010.zip › 8. KEGG Enrichment Analysis/bubble_before.pdf]

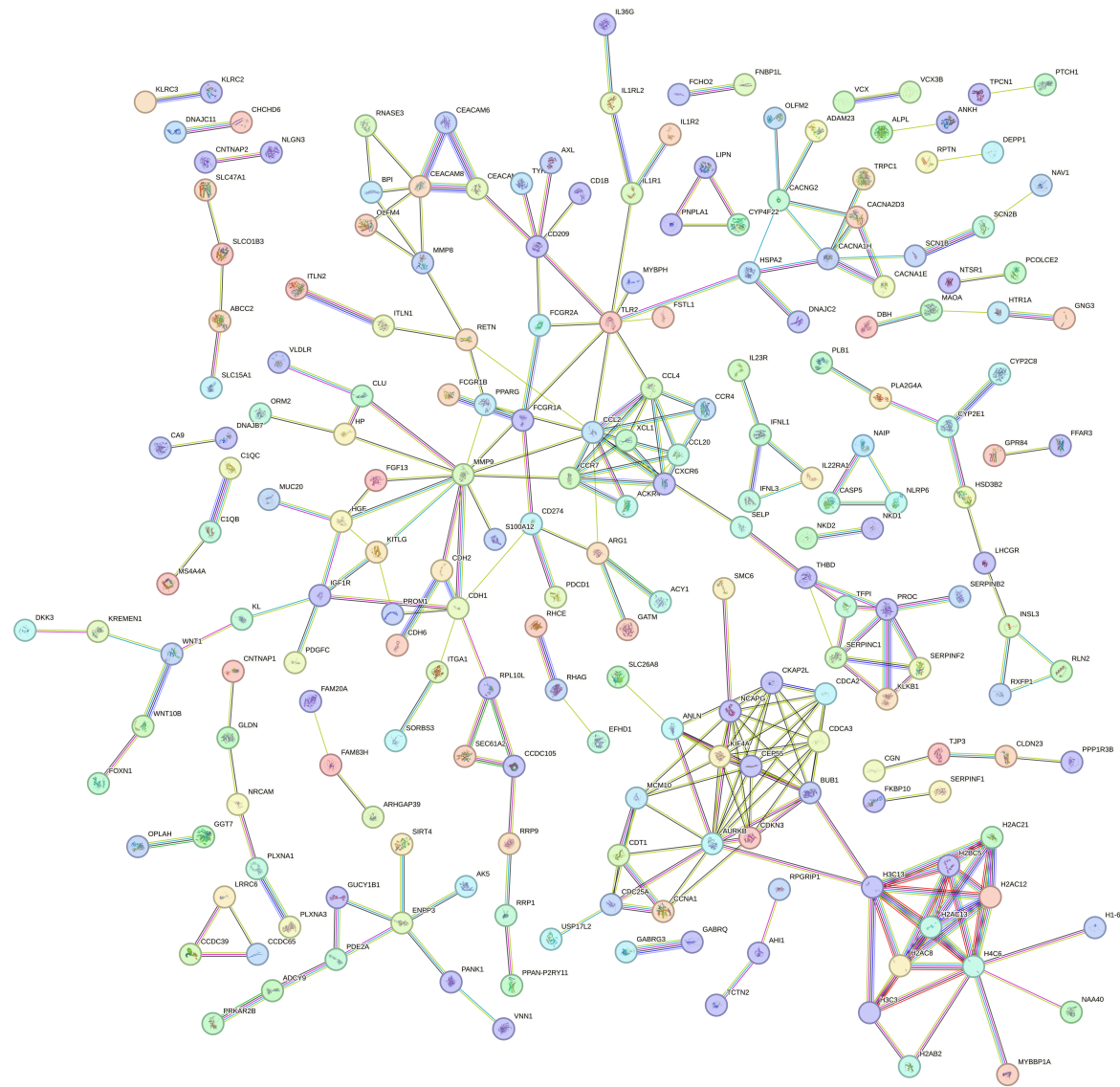

Supplement: S10 File — (ZIP) [file pone.0324337.s012.zip › 10. PPI/string_hires_image.pdf]

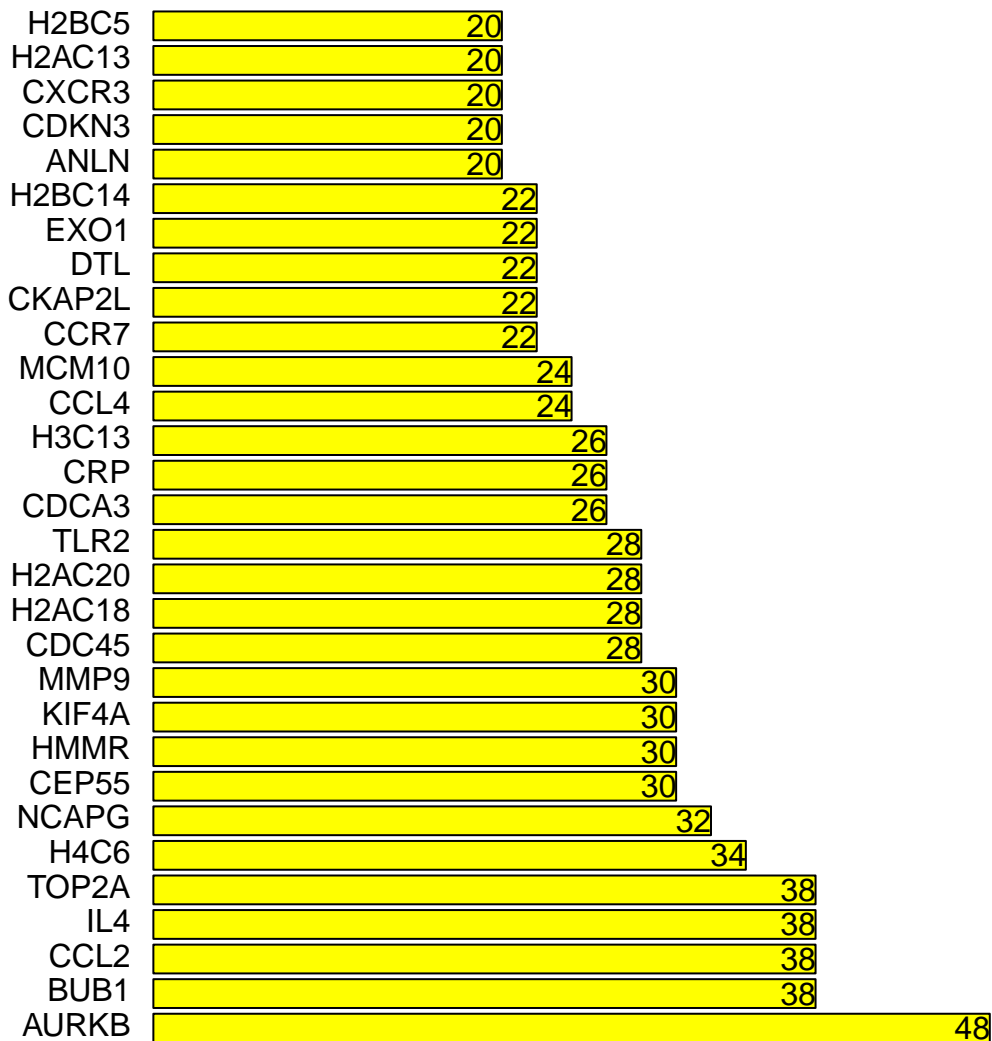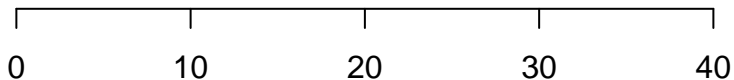

Supplement: S11 File — (ZIP) [file pone.0324337.s013.zip › 11. Hub Genes/barplot.pdf]

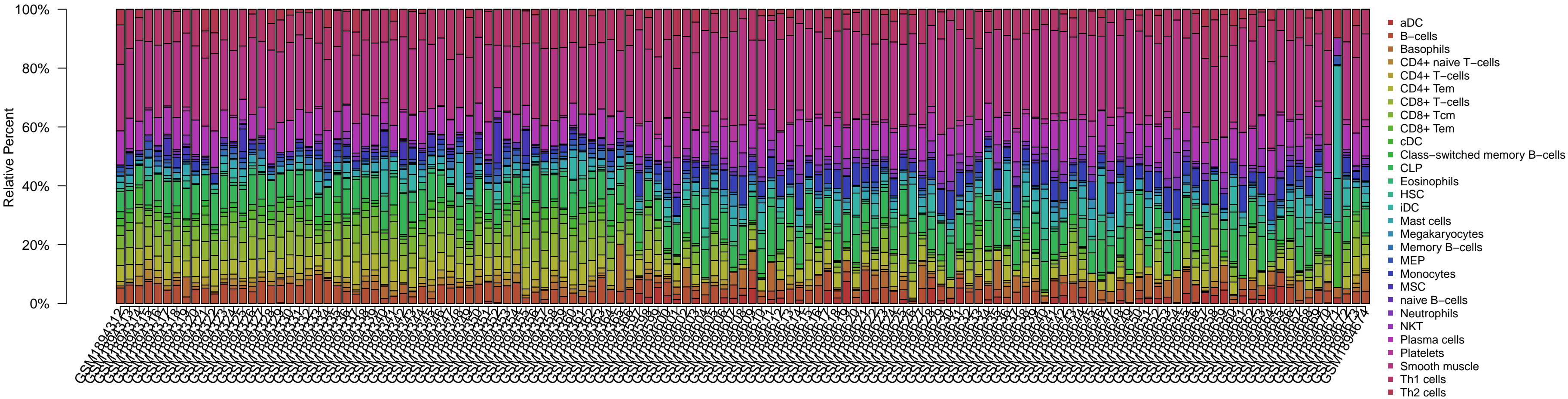

Supplement: S15 File — (ZIP) [file pone.0324337.s017.zip › 4. Bar Chart/barplot.pdf]

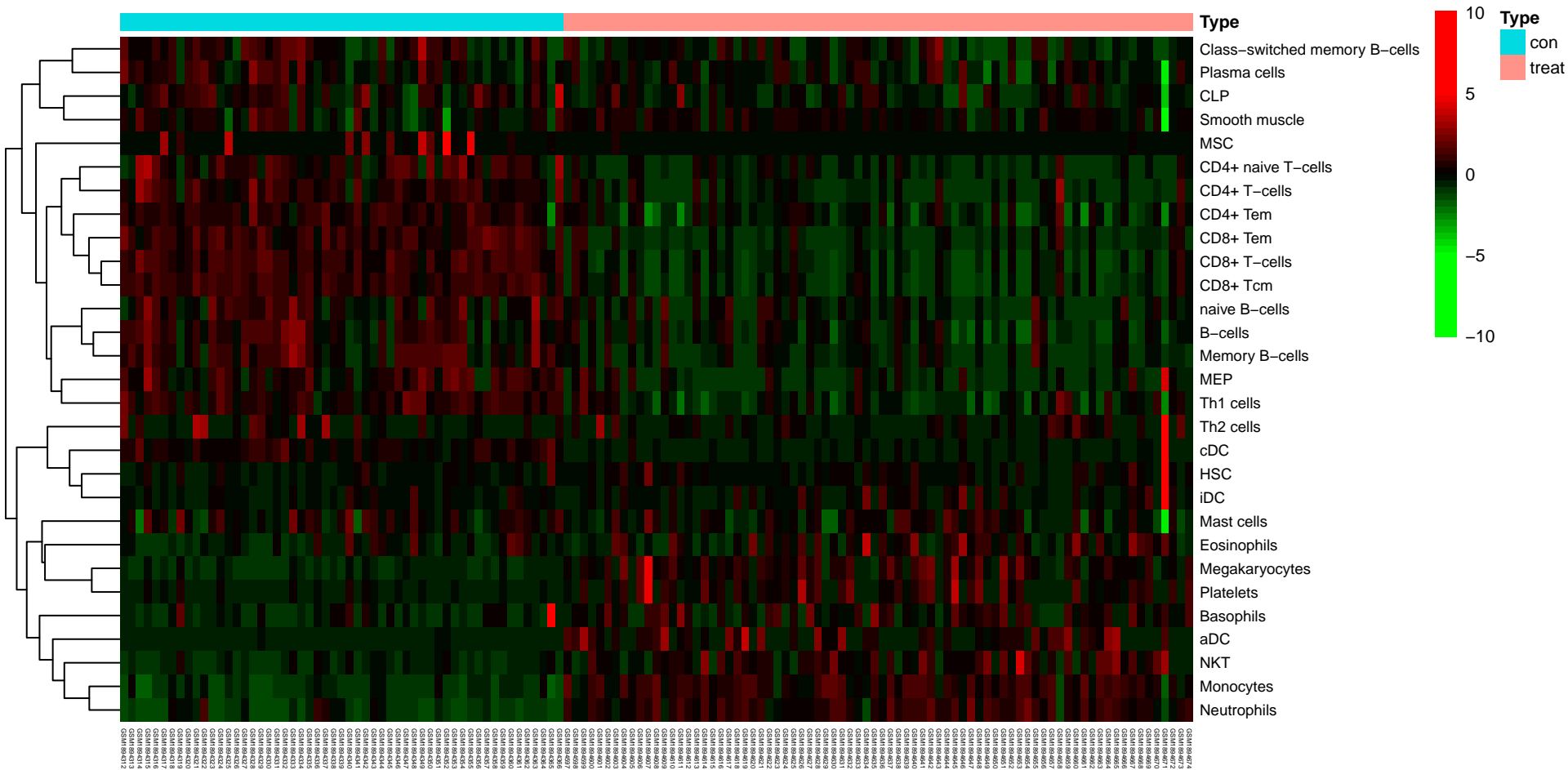

Supplement: S16 File — (ZIP) [file pone.0324337.s018.zip › 5. Heatmap/heatmap.pdf]

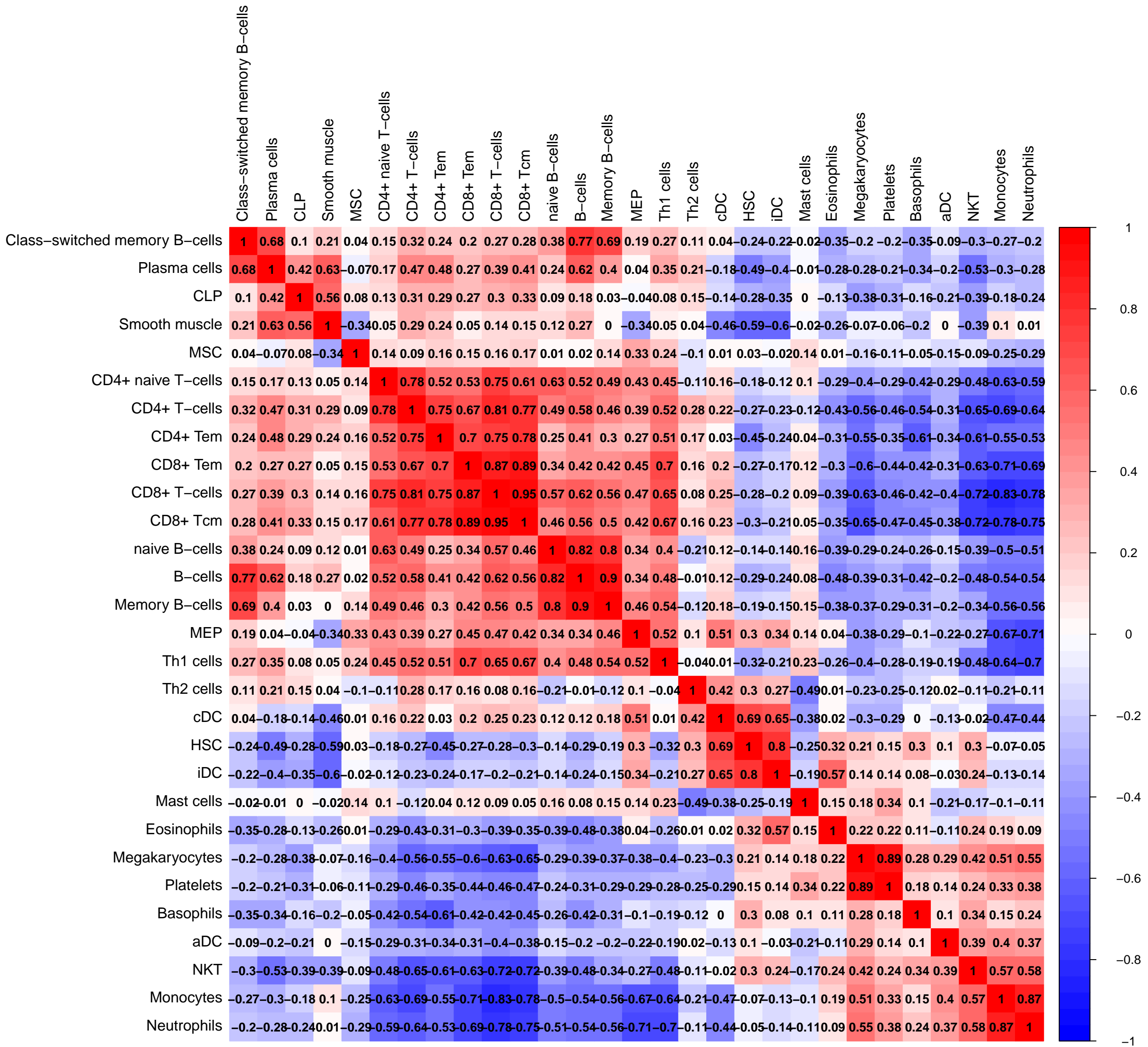

Supplement: S17 File — (ZIP) [file pone.0324337.s019.zip › 6. Correlation Heatmap/corHeatmap.pdf]

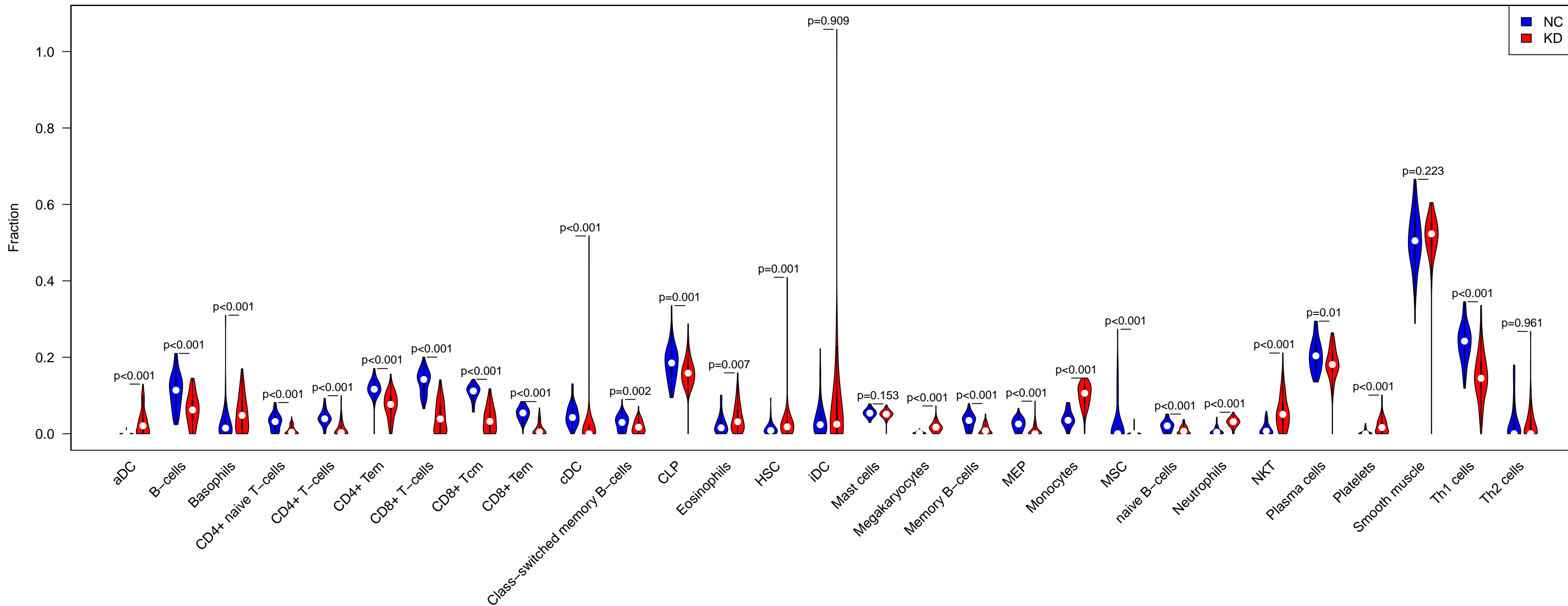

Supplement: S18 File — (ZIP) [file pone.0324337.s020.zip › 7. Violin Plot/vioplot.pdf]

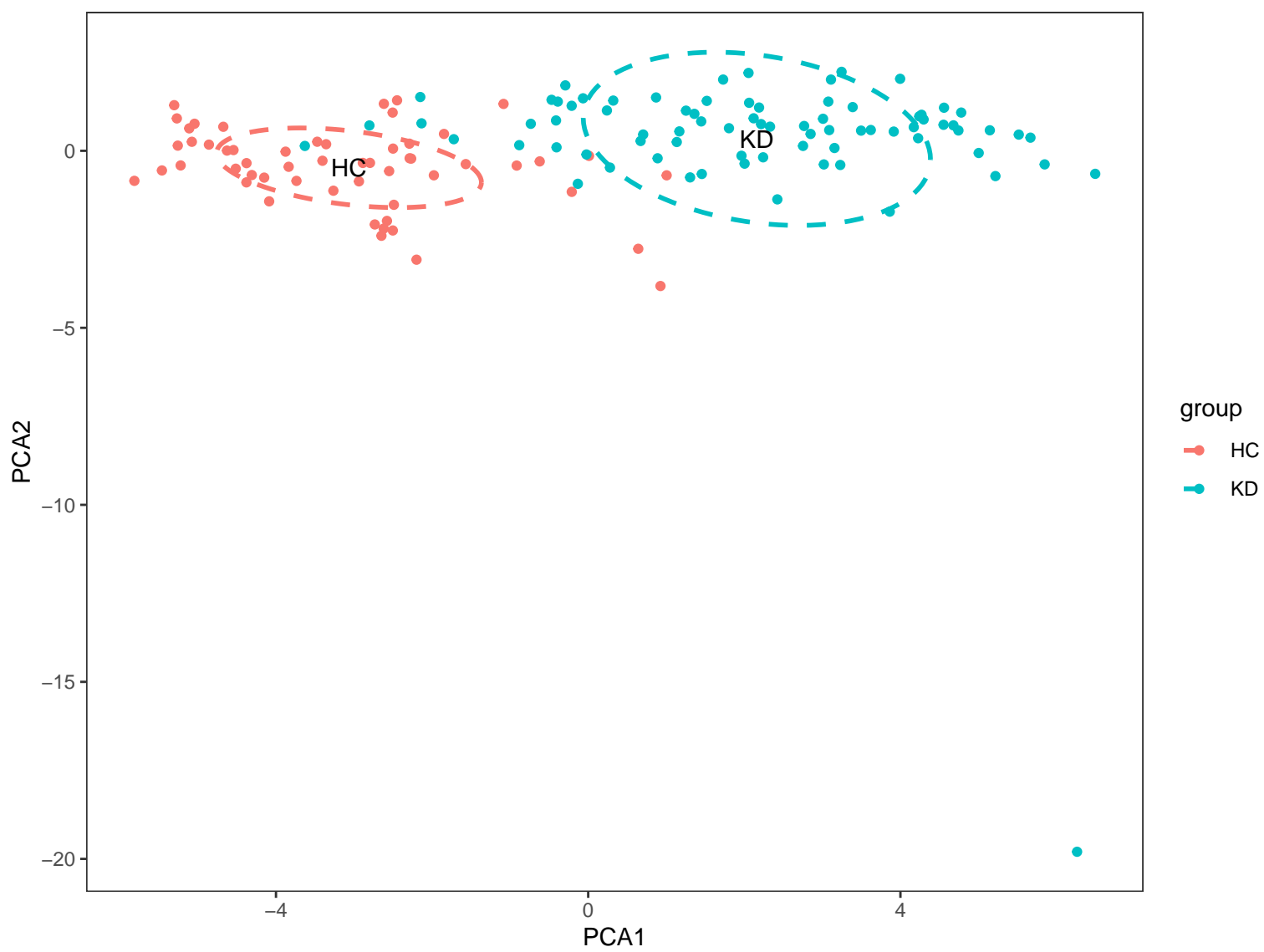

Supplement: S19 File — (ZIP) [file pone.0324337.s021.zip › 8. PCA/PCA.pdf]

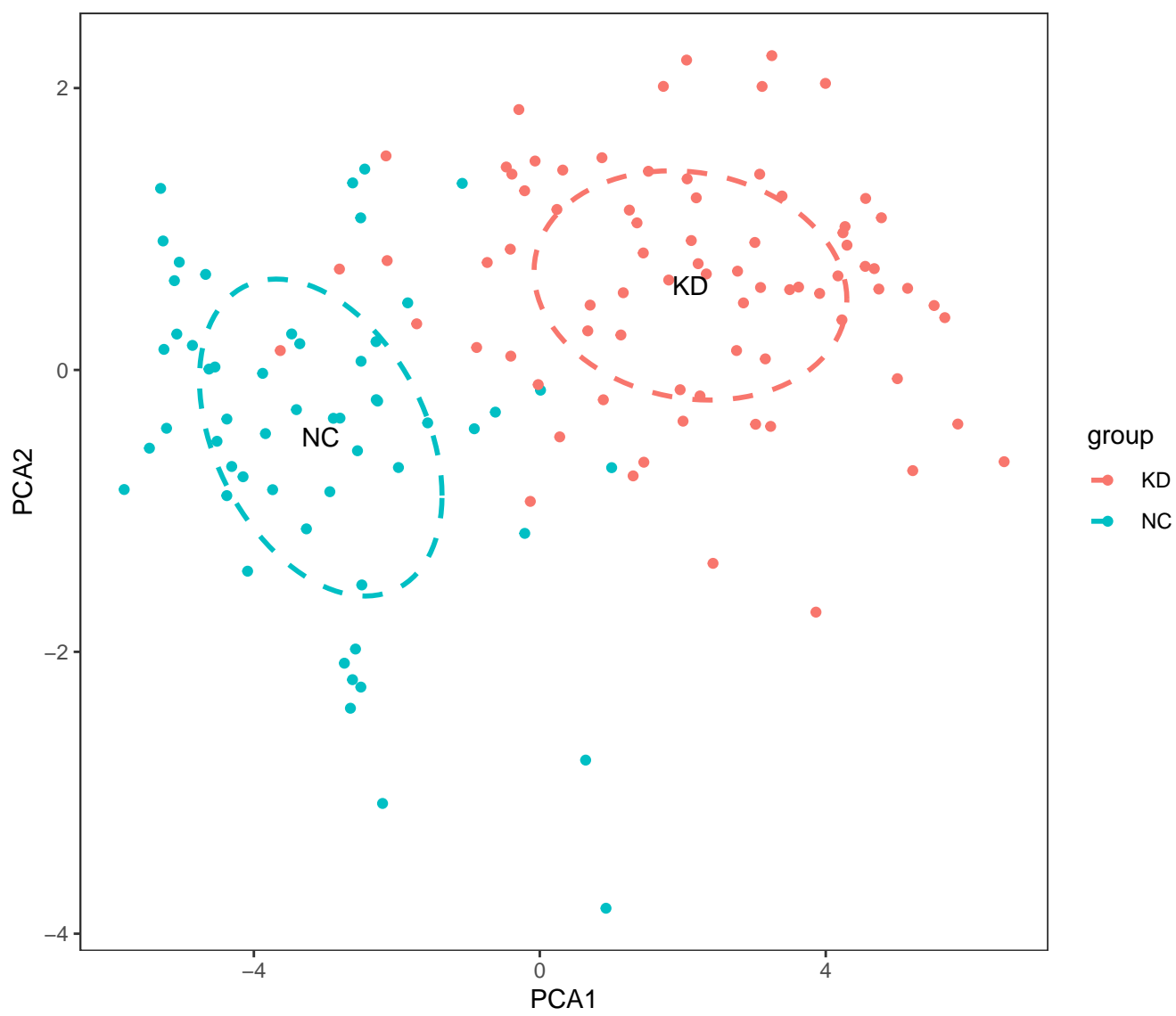

Supplement: S19 File — (ZIP) [file pone.0324337.s021.zip › 8. PCA/PCA_modified.pdf]

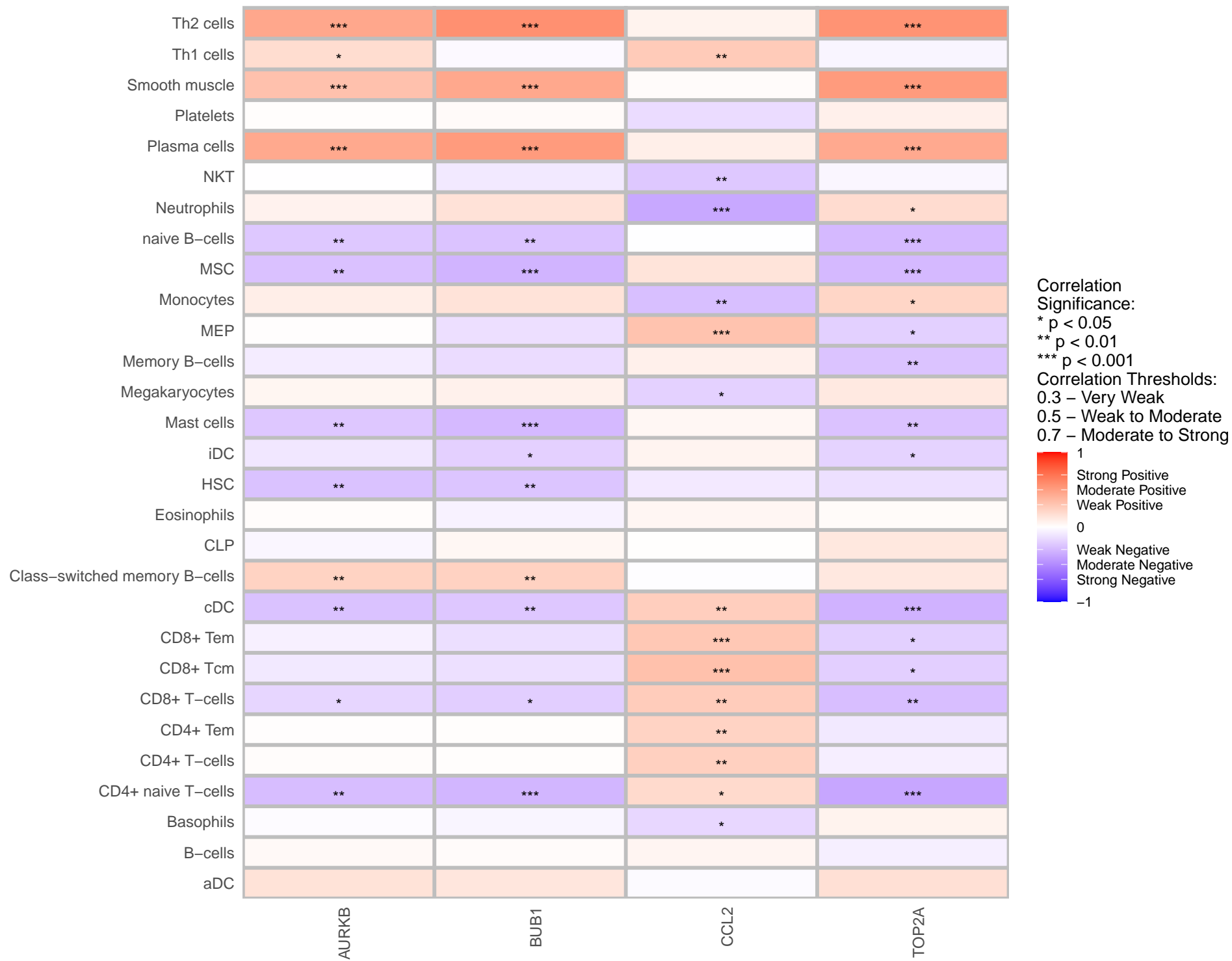

Supplement: S20 File — (ZIP) [file pone.0324337.s022.zip › 9. Immune Cell Correlation Analysis/cor.pdf]

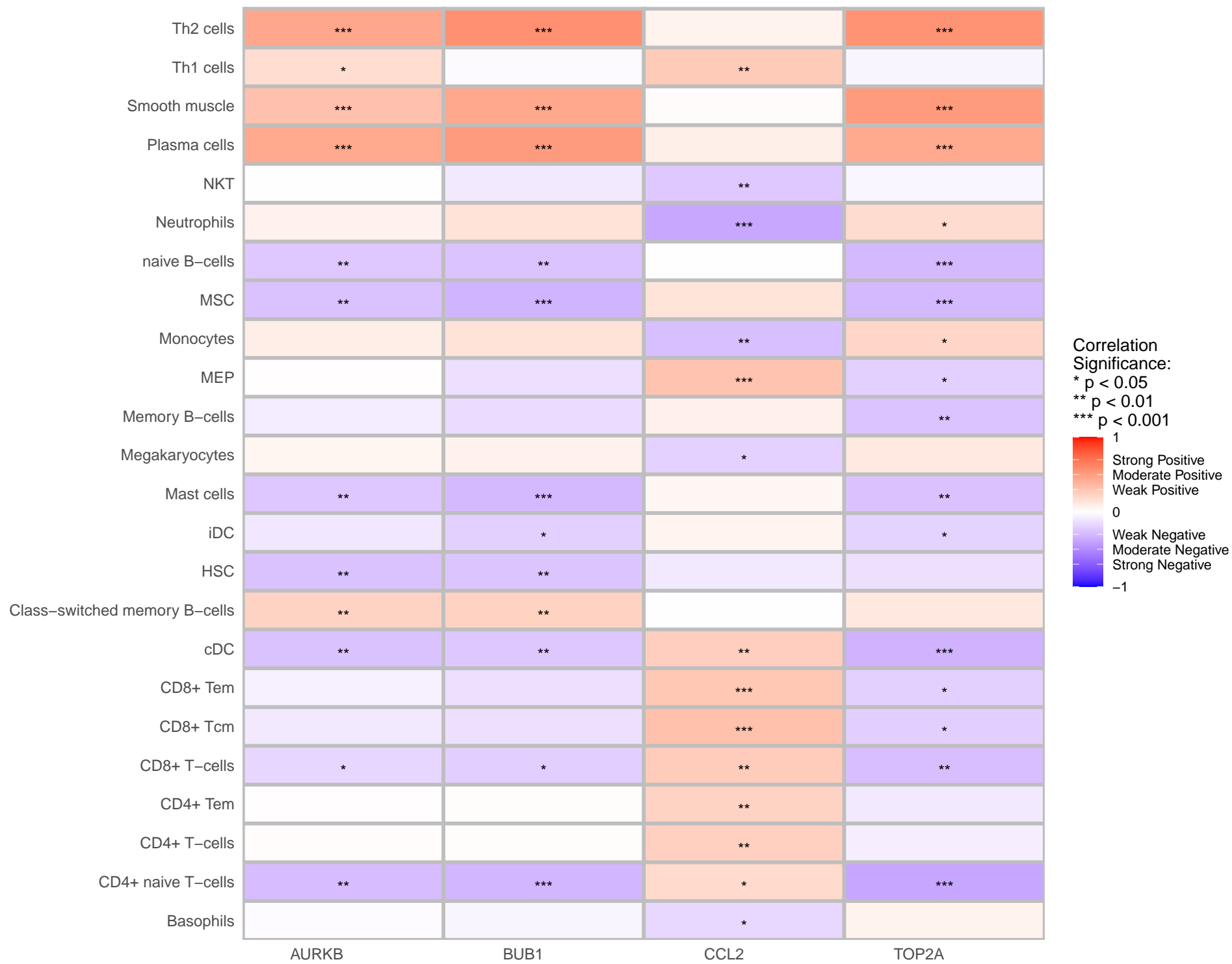

Supplement: S20 File — (ZIP) [file pone.0324337.s022.zip › 9. Immune Cell Correlation Analysis/cor_filtered.pdf]
