# Supplementary material for: Identification of potential biomarkers associated with immune cell infiltration patterns in Kawasaki disease via bioinformatics
Source: PLoS One. 2025 Jun 2;20(6):e0324337. doi: 10.1371/journal.pone.0324337 (PMC12129191; doi:10.1371/journal.pone.0324337)
Supplement: S9 File — (ZIP) [file pone.0324337.s011.zip › 9. KEGG Diagram/circ.pdf]

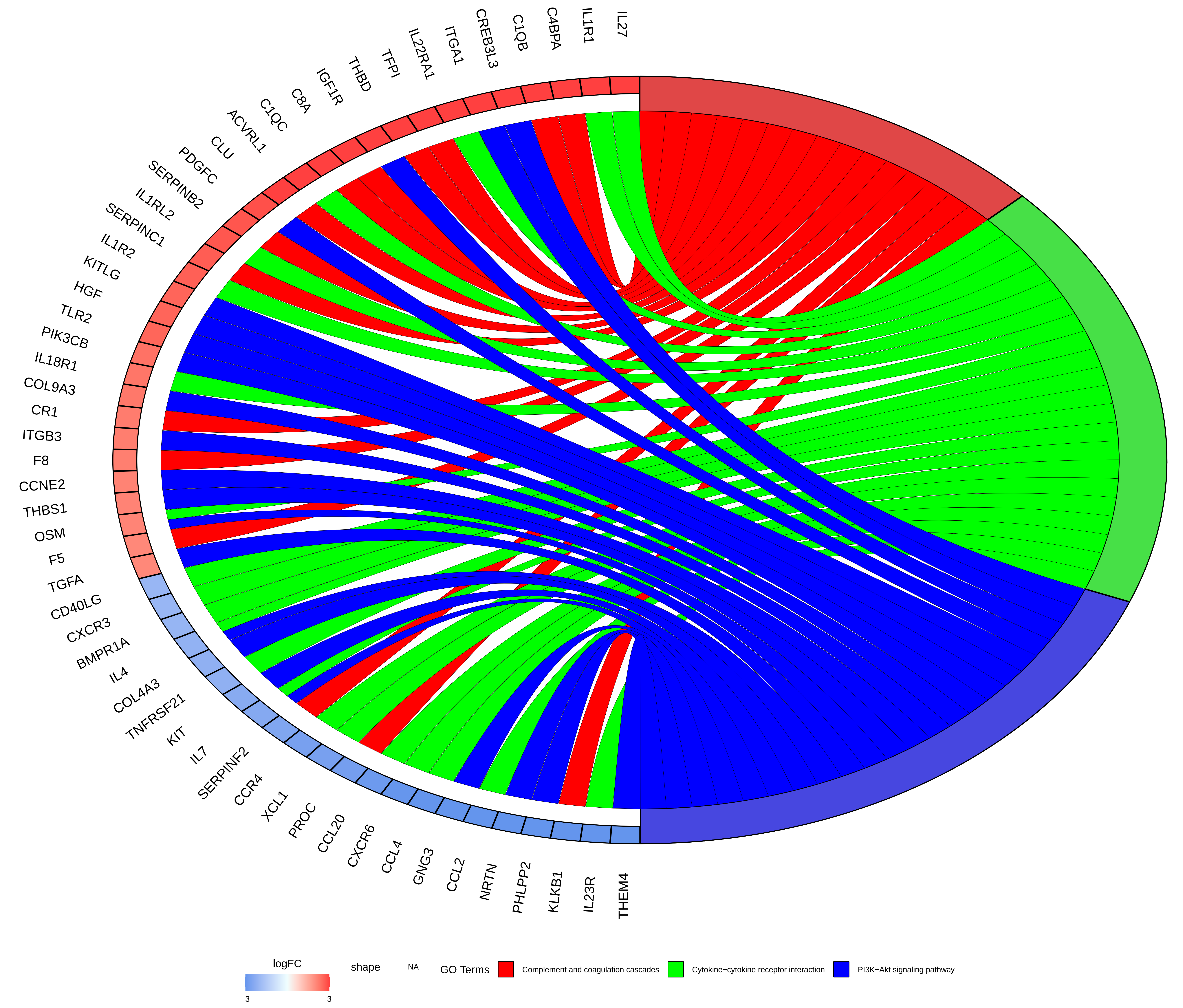
IL27
IL1R1
C4BPA
C1QB
CREB3L3
ITGA1
IL22RA1
TFPI
THBD
IGF1R
C8A
C1QC
ACVRL1
CLU
PDGFC
SERPINB2
IL1RL2
SERPINC1
IL1R2
KITLG
HGF
TLR2
PIK3CB
IL18R1
COL9A3
CR1
ITGB3
F8
CCNE2
THBS1
OSM
F5
TGFA
CD40LG
CXCR3
BMPR1A
IL4
COL4A3
TNFRSF21
KIT
IL7
SERPINF2
CCR4
XCL1
PROC
CCL20
CXCR6
CCL4
GNG3
CCL2
NRTN
PHLPP2
KLKB1
IL23R
THEM4
logFC
−3
3
shape
NA
GO Terms
Complement and coagulation cascades
Cytokine−cytokine receptor interaction
PI3K−Akt signaling pathway
