# Supplementary material for: Identification of potential biomarkers associated with immune cell infiltration patterns in Kawasaki disease via bioinformatics
Source: PLoS One. 2025 Jun 2;20(6):e0324337. doi: 10.1371/journal.pone.0324337 (PMC12129191; doi:10.1371/journal.pone.0324337)
Supplement: Attachment — Submitted filename: Response to reviewers.docx [file pone.0324337.s024.docx]

Revised Submission PONE-D-24-55722: Identification of Potential Biomarkers associated with Immune Cell Infiltration Patterns in Kawasaki Disease via Bioinformatics

Dear Dr. Tan and the Editorial Team,

Thank you very much for the detailed and constructive comments from Reviewer #1, Reviewer #2, Reviewer #3, and Reviewer #4 on our manuscript. We have taken these suggestions very seriously and have made substantial revisions to address the issues raised, with particular attention to the clarity, methodology, and relevance of our findings. Here is a summary of our responses:

**Reviewer #1:**  Thank you for your invitation.
The manuscript is about the Identification of AURKB , BUB1 , CCL2 , and TOP2A as biomarkers, and immune infiltration in kawasaki disease through bioinformatics analysis. It is of certain value, the structure is clear and the charts are appropriate, but please consider some details as outlined below:

Response:

Thank you for your positive feedback and for acknowledging the value, clarity of structure, and appropriateness of the charts in our manuscript. We have updated the title to more accurately reflect the content of our study, which is now titled "Identification of Potential Biomarkers associated with Immune Cell Infiltration Patterns in Kawasaki Disease via Bioinformatics."

We have carefully considered your comments and have made the following revisions to address your points:

**Comment 1:** Please check through the manuscript for writing. There are differences in the use of statistical notation throughout the manuscript. "p" in line 74 and 78 should be italicized, and in line 99 presented as "p-values". In line 87, "xCell" should be capitalized as "XCell", etc.

**Response:**
Thank you for your thorough review and for bringing these important points to our attention. We have carefully reviewed the manuscript and made the necessary corrections to ensure consistency and accuracy in our use of statistical notation and terminology throughout the document. Specific Corrections Made:

--The "p" on original lines 74 and 78 was italicized (see lines 65 and 71 now).

--We have replaced "p-values" with "*p*" on the line that was originally 99 (see line 95 now) to provide clarity and adhere to proper terminology.

--The term "xCell" has been correctly capitalized as "XCell" in the sentence that was originally on line 87 (see line 81 now).

**Comment 2:** When using GO and KEGG to identify the enriched functions and pathways, how do you handle the issue of functional redundancy to prevent over-interpretation caused by a single gene's involvement in multiple pathways?
**Response:**

Thank you for your insightful comments and for highlighting the need to address the issue of functional redundancy in our analysis using GO and KEGG. We appreciate your concern regarding the potential over-interpretation caused by a single gene's involvement in multiple pathways. To handle redundancy, we set p-value < 0.05 and q-value < 0.4, and defined a 0.7 overlap threshold to merge highly overlapping pathways. We selected the most significant pathway (lowest p-value) as the representative when redundancy was detected. These parameters were applied consistently across both analysis to ensure uniform redundancy handling (see line 68 to 71 now).

**Reviewer #2:** This manuscript presents a bioinformatics study that identifies potential biomarkers (AURKB, BUB1, CCL2, and TOP2A) and investigates immune cell infiltration patterns in Kawasaki disease (KD). The study offers valuable insights into the molecular mechanisms underlying KD and provides evidence for the potential use of these biomarkers in diagnosis and targeted therapy. However, there are several areas where the manuscript could be improved in terms of structure, clarity, language, and scientific depth. (1)The manuscript lacks a clear, cohesive structure in some sections, particularly in the Results and Discussion. There is frequent overlap, repetition, and mixing of topics;(2) The manuscript contains frequent grammatical errors, awkward phrasing, and informal expressions, which reduce its readability and professionalism. (3)Many ideas, such as the roles of AURKB and BUB1 in mitosis or the enrichment of immune-related GO terms, are repeated across sections without adding new insights. This leads to redundancy. (4) While the manuscript provides detailed descriptions of hub gene functions and pathways, the relevance to KD is not consistently emphasized. Discussions about cancer-related roles of AURKB, BUB1, and TOP2A dominate, while their specific roles in KD are underexplored. Below are partial problems listed in the order they are stated in the paper.

**Response:**

Thank you for your constructive feedback. We have revised the manuscript as follows: (1) Structure and Coherence: We have reorganized the Results and Discussion sections to ensure clarity and logical flow, removing redundancy and integrating related topics; (2) Language and Grammar: The manuscript has been polished to correct errors and improve readability; (3) Redundancy: We have streamlined repetitive content, focusing discussions on the genes' roles in KD rather than their functions in cancer; (4) Relevance: We have added detailed discussions on the specific roles of AURKB, BUB1, CCL2, and TOP2A in KD, supported by the latest research literature. Below are our responses to the partial problems in the order they are stated.

**Comment 1:** Use Greek letters consistently (e.g., TNF-α instead of TNF-a) to adhere to academic conventions.

**Response:**

Thank you for pointing out the need for consistency in the use of Greek letters. We have thoroughly reviewed the manuscript and made the following specific changes: All instances of "TNF-a" have been revised to "TNF-α" to ensure consistency with academic conventions.

**Comment 2**: "However, patients do no show apparent clinical manifestations, thus delaying diagnosis and treatment."better changes to "However, KD patients often lack apparent clinical manifestations, leading to delayed diagnosis and treatment."

**Response:**

Thank you for your valuable suggestion regarding the clarity and accuracy of the sentence. We agree with your suggestion and have revised the sentence accordingly. The revised sentence now reads:" However, KD patients often lack apparent clinical manifestations, leading to delayed diagnosis and treatment (see lines 34 to 35 now)."

**Comment 3**: Some expressions could be made more concise and formal to enhance the academic tone of your writing."The data were cleaned using Limma11packages in R software (4.3.0)." , it could be better revised as "Data normalization and preprocessing were performed using the limma package in R (version 4.3.0)."

**Response:**
Thank you for your suggestion on improving the conciseness and formality of our writing. We agree with your suggestion and have revised the sentence accordingly. The revised sentence now reads: "Data preprocessing was performed using the limma package in R (version 4.3.0) (see lines 60 to 61 now)"

**Comment 4**: "log fold change" should be clarified as "log2 fold change" to be consistent with standard terminology.

**Response:**
Thank you for your suggestion regarding the clarification of terminology. We agree with your suggestion and have clarified "log fold change" as "log2 fold change" throughout the manuscript to ensure consistency with standard terminology (see lines 65, 95 and 96 now).

**Comment 5:** The current structure is mostly fine, but for better readability and organization,

please start each subsection with a brief explanation of the purpose of the analysis. For example:In 2.2, briefly state the purpose of constructing the PPI network (e.g., to identify interactions between differentially expressed genes), etc.

**Response:**

Thank you for your suggestion on improving the readability and organization of our manuscript. We have revised the manuscript to include a brief explanation of the purpose of each analysis at the beginning of each subsection. For example:

--The purpose of screening DEGs: To identify genes with significant expression changes under different conditions (see lines 60 to 61 now).

--The purpose of GO and KEGG enrichment analysis: To achieve functional interpretation of gene clusters (see lines 68 now).

--The purpose of constructing the PPI network: To identify interactions among DEGs (see lines 73 now).

--The purpose of hub genes: To identify key genes with significant interactions in biological networks (see lines 73 now).

--The purpose of Cell Infiltration analysis: The XCell algorithm was then used to evaluate the infiltration scores of 29 immune and non-immune cells (see lines 81 to 82 now).

--The purpose of Correlation analysis and PCA: To assess the correlation between hub genes and immune cells, and to assess the efficacy of a node-gene signature in distinguishing KDs from NCs (see lines 86 to 87 now).

**Comment 6:** Some expressions are too general or lack specificity. Consider the following refinements:

2.2: PPI Network Construction: Specify the STRING database threshold (e.g., combined score cutoff for significance); Explain how hub genes were identified (e.g., via centrality metrics like degree or betweenness). Include the version of Cytoscape for reproducibility.

**Response:**

Thank you for your valuable feedback regarding the specificity of our expressions in Section 2.2. We have made the following refinements to address your concerns:

--STRING Database Threshold: We have specified the STRING database threshold as ≥0.7 in the revised text (see lines 70 now);

--Hub Gene Identification: We have clarified that hub genes were identified as those with degree centrality above the 90th percentile in the PPI network, using the igraph R package (see lines 75 to 77 now);

--Cytoscape Version: We thank the reviewer for raising this point. Upon further review, we realized that Cytoscape was mentioned in the manuscript but was not actually used in our study. To ensure accuracy and reproducibility, we have removed all references to Cytoscape and clarified the tools we actually employed. We apologize for any confusion this may have caused.

2.3: Immune Cell Infiltration Analysis: Shorten and simplify the description of xCell without losing its technical essence. State how input data were normalized or preprocessed before applying xCell. Reorganize for better readability: explain the analysis, significance threshold, and visualization in sequence.

**Response:**

Thank you for your suggestions regarding Section 2.3 on Immune Cell Infiltration Analysis. We have revised the section to address your concerns as follows:

--Shortened and Simplified Description of xCell: We have shortened and simplified the description of the XCell algorithm while retaining its technical essence. The revised sentence now reads: "We then used the XCell algorithm to evaluate infiltration scores for 29 immune and non-immune cells" (see lines 81 to 82 now).

--Normalization and Preprocessing of Input Data: To address the reviewer's concerns, we have normalized and preprocessed the GSE73461 dataset by removing genes with zero average expression, applying scaling normalization, and excluding IL4 due to low post-normalization expression (see lines 79 to 80 now).

--Reorganized for Better Readability: We have reorganized the text to improve readability by sequentially explaining the analysis, significance threshold, and visualization. The revised text now clearly outlines the steps: data preparation and normalization, application of the XCell algorithm to evaluate infiltration scores, identification of significant differences at P < 0.05, and visualization using heatmaps and violin plots (see lines 79 to 84 now).

2.4: Correlation Analysis and PCA: Specify "hub genes" and "immune cells" explicitly in the correlation analysis description. Use consistent package names (e.g., "ggplot2" in backticks). For Spearman analysis: indicate how correlation coefficients were interpreted (e.g., thresholds for "strong" or "weak" correlations).

**Response:**

Thank you for your detailed feedback regarding Section 2.4. We have made the following revisions to address your concerns:

--Explicit Specification of "Hub Genes" and "Immune Cells": We have revised the description to explicitly specify “hub genes” and “immune cells” in the correlation analysis. The updated text now reads: “To evaluate the correlation between candidate hub genes and infiltrating immune cells, and to assess the efficacy of a node-gene signature in distinguishing KDs from NCs, we used the Spearman correlation method (P < 0.05)” (see lines 86 to 88 now). We acknowledge that the XCell algorithm includes both immune and non-immune cells, but our analysis specifically focused on immune cells in this context.

--Consistent Package Names: We have standardized the formatting of package names throughout the manuscript, using backticks for consistency (e.g., ggplot2) (see lines 89 and 91 now). Thank you for pointing this out.

--Interpretation of Correlation Coefficients: We have added a description of how correlation coefficients were interpreted in the Spearman analysis. The thresholds are now clearly defined as follows: strong (|r| ≥ 0.7), moderate (0.4 ≤ |r| < 0.7), and weak (0.1 ≤ |r| < 0.4) (see lines 88 to 89 now).

**Comment 7:** The logical flow of the results section is unclear, and the connections between subsections are weak and the results feel fragmented, and there is no clear summary of key findings in some sections. Please reorganize the results section to create a more coherent flow. Use a logical order such as:

--Identification of DEGs: Provide the results of DEG analysis and briefly explain its significance.

--GO and KEGG Enrichment Analysis: Highlight the biological relevance of the enriched pathways and terms.

--PPI Network Construction and Hub Gene Identification: Emphasize the key findings related to hub genes.

--Immune Cell Infiltration Analysis: Explain the immune landscape differences in KD.

--Correlation Analysis and PCA: Highlight the relationships between immune cells and hub genes, and show how PCA distinguishes KD and NC samples.

**Response:**

Thank you for your valuable feedback regarding the logical flow of the results section. We have thoroughly reorganized the Results section to improve logical flow and coherence. We have structured the subsections in a more logical order and added transitions to strengthen the connections between them. Additionally, we have included clear summaries of key findings in each section to provide a better overview of the results. Thank you for your valuable suggestions. The specific changes are as follows:
--Identification of DEGs: We have revised the section on DEG identification to provide more detailed results and explain their significance. The updated text now reads: “In this study, we identified 1229 DEGs with P-values < 0.05 and |log2 FC|≥ 2, including 720 up-regulated and 509 down-regulated genes. Notably, those with |log2 FC| > 8 are particularly important for understanding specific biological processes or disease states and require further study (Fig. 1)” (see lines 95 to 97 now).

--GO and KEGG Enrichment Analysis: We have revised the section on GO and KEGG enrichment analysis to better highlight the biological relevance of the enriched pathways and terms. The updated text now emphasizes how these findings relate to Kawasaki disease (KD) and their significance in understanding its molecular mechanisms. Specifically, we discuss the links to acute inflammatory response and plasma membrane components in GO analysis, as well as the enrichment in cytokine interactions and PI3K-Akt signaling in KEGG analysis. These results support KD's known inflammatory and immune-mediated nature and provide insights into potential therapeutic targets (see lines 99 to 106 now).
--PPI Network Construction and Hub Gene Identification: We have revised the section on PPI network construction and hub gene identification to emphasize the key findings related to hub genes. The updated text now clearly highlights the identification of five prominent candidate hub genes (AURKB, BUB1, CCL2, IL4, and TOP2A) and their significant expression levels, underscoring their pivotal role in KD pathogenesis (see lines 108 to 112 now).

--Immune Cell Infiltration Analysis: We have revised the section on immune cell infiltration analysis to explain the immune landscape differences in KD. Employing the XCell algorithm, we quantified 29 cell types and found that KD is characterized by decreased CD4+ T cells, CD8+ T cells, B cells, and Th1 cells, while Megakaryocytes, platelets, basophils, aDCs, NKT cells, Monocytes, and neutrophils are increased compared to NC. We also identified key correlations between these cell types and used PCA to highlight immune dysregulation in KD (see lines 114 to 122 now). We have included a PCA plot (Fig S2D) that effectively distinguishes KD samples from NC samples, demonstrating the distinct immune profiles between the two groups (see lines 121 to 122 now).

--Correlation Analysis: We have revised the correlation analysis section to highlight the relationships between immune cells and hub genes. The updated text now clearly shows how key hub genes (AURKB, BUB1, TOP2A, and CCL2) are correlated with various immune cells in KD. Specifically, AURKB, BUB1, and TOP2A are linked to Th1 cells and Plasma cells, while CCL2 shows complex interactions with T-cell subsets and other immune cells. These relationships underscore the intricate immune dynamics in KD (see lines 126 to 130 now).

**Comment 8:** Replace vague phrases like "immune infiltration" with "immune cell infiltration scores."

**Response:**

Thank you for your suggestion. We have revised the manuscript to replace vague phrases like "immune infiltration" with more specific terms such as "immune cell infiltration scores" to enhance clarity and precision (see line 12 now).

**Comment 9:** Section 3.3 is better suited as a methods description rather than a presentation of results.
**Response:**

Thank you for your comment regarding Section 3.3. We have restructured the Section to serve as a presentation of results, ensuring that the methodology is clearly described in the Methods section and the findings are presented in the Results section. The revised section now reads: "To pinpoint critical genes contributing to Kawasaki disease (KD), we constructed a PPI network. Nodes of 876 intersecting genes were detected in the PPI network using a medium confidence level of ≥0.7 (Fig 2E). Our analysis using an R package reveals five prominent candidate hub genes: AURKB, BUB1, CCL2, IL4, and TOP2A (Fig 2F). These genes, central to the network's structure, exhibit significant expression levels, underscoring their pivotal role in KD pathogenesis (see lines 108 to 112 now).

**Comment 10:** In Fig. 3E, the bottom-right point in the PCA plot may be treated as an outlier and removed before further analysis.

**Response:**

Thank you for your suggestion regarding the PCA plot. We have carefully examined the bottom-right point in the original Figure 3E, which is now Supplementary Figure 2D, and agree that it appears to be an outlier. We have removed this data point from the analysis and re-run the PCA to ensure that the results are not skewed by this outlier.

**Comment 11:** The discussion lacks a clear structure, making it difficult to follow the key findings and their implications. There is no clear progression from specific results to broader implications. Multiple ideas (e.g., hub gene functions, immune mechanisms, PI3K-Akt pathway) are mixed within the same paragraph without proper separation, which makes the discussion overwhelming and repetitive. Please restructure the discussion into clearly defined subsections for better readability.
**Response:**

Thank you for your valuable feedback regarding the structure of the discussion section. We have restructured the Discussion into clearly defined subsections to improve readability and clarity:

--KD Background and Significance: Briefly describe KD background and the importance of this study (see lines 132 to 137 now).

--DEGs and Pathway Analysis: Discuss DEGs' enrichment in functional and pathway (see lines 138 to 162 now).

--Hub Gene Functions: Analyze the roles of hub genes (AURKB, BUB1, TOP2A, CCL2) in cell cycle, inflammation, and tissue repair (see lines 163 to 178 now).

--Immune Cell Infiltration Analysis: Examine changes in cell infiltration in KD samples and their effects on KD progression (see lines 179 to 192 now).

--Correlation between Hub Genes and Immune Cells: Explore relationships between hub genes and immune cells and their potential applications in KD (see lines 193 to 204 now).

**Comment 12:** The discussion sometimes strays from Kawasaki disease (KD) and focuses too much on general roles of genes (e.g., their roles in tumor progression or mitosis). While these are important, the relevance to KD is not always clearly established. For example, the discussion on IL-4 mentions IVIG studies in mice but does not sufficiently connect these findings to KD in humans.

**Response:**

Thank you for your insightful comment regarding the focus of the discussion. We have revised the discussion section to focus on the relevance of our findings to Kawasaki Disease (KD). We removed references to IL-4, which was excluded from our analysis due to low expression after normalization. The revised text now clearly links the identified genes (AURKB, BUB1, TOP2A, and CCL2) to KD pathology, emphasizing their roles in cell cycle regulation, inflammation, and tissue repair. We have also expanded on the immune cell infiltration patterns observed in KD samples and clarified the correlations between hub genes and immune cells. These changes ensure that the discussion remains focused on KD-specific findings and their implications for understanding and treating the disease.

**Comment 13:** "It can be concluded that the four-gene above mentioned take part in the course of immune cell-mediated development in KD patients." (grammatical and awkward phrasing)
**Response:**

Thank you for your insightful comments. We have carefully revised the sentence in question to improve its clarity and grammatical accuracy. The original sentence has been modified to: "All of these show that AURKB, BUB1, TOP2A, and CCL2 play key roles in KD through affecting cell functions and inflammation response, and may aid in coronary artery repair, making them important for treatment (see lines 202 to 204 now)."

**Comment 14:** The conclusion is too brief and does not adequately summarize the key findings or their broader implications. Please provide a stronger, more detailed conclusion that highlights the study’s key contributions and potential impact:
--Recap the four hub genes and their potential as diagnostic biomarkers.
--Emphasize the unique immune cell infiltration patterns identified in KD.
--Discuss how the findings provide a foundation for future research into KD mechanisms and therapeutic strategies.

**Response:**

Thank you for your feedback. We have revised the conclusion to better summarize our key findings and their implications:

--We identified four key genes (AURKB, BUB1, TOP2A, and CCL2) that may serve as diagnostic biomarkers for KD.

--We discovered unique immune cell infiltration patterns in KD, providing new insights into its immunopathology.

--These findings suggest new treatment targets and lay the foundation for future research into KD mechanisms and therapies. Further validation in larger studies is needed.

The revised conclusion reads as follows: "This study identified four key genes—AURKB, BUB1, TOP2A, and CCL2—that are involved in KD and could serve as diagnostic biomarkers. We also discovered unique immune cell patterns that offer new insights into KD's immunopathology. These genes, interacting with immune cells and linked to the PI3K/AKT and CCRI pathways, present new targets for KD treatment. While these findings are promising, they require further validation in larger studies and deeper investigation to develop effective clinical applications for KD (see lines 206 to 210 now)."

**Reviewer #3:** In this study, the authors looked at up- and downregulated genes in Kawasaki disease (KD) patients compared to healthy patients. They clustered the genes by pathways revealing immune system-related processes, as expected in KD, and PI3K-Akt signaling as potentially important for KD. Further protein-protein interaction analysis led to the identification of AURKB, BUB1, CCL2, IL4 and TOP2A as potential protein biomarkers for KD. After looking at the genes, they looked at the immune cell distribution suggesting that innate immune cells are more present in patients with KD, but adaptive immune cells are less present. They ended the study by using PCA to look at relationship between immune cell levels and gene expression levels for the previously identified ones potentially involved in KD. While identifying biomarkers for KD is of great need due to the lack of KD-specific biomarkers at the moment, the reviewer believes that this study lacks discussion and critical analysis of the results.
**Response:**

Thank you for your insightful comments. We have carefully revised our manuscript to address your concerns regarding the need for more discussion and critical analysis of our results. Here are the key enhancements:

--Enhanced Discussion of Biomarkers: We have expanded the discussion to critically evaluate the potential of the identified hub genes (AURKB, BUB1, TOP2A, and CCL2) as biomarkers for KD. We have analyzed their roles in inflammation, immune response, and tissue repair, and how they may impact KD pathogenesis (see lines 163 to 178 now).

--Critical Analysis of Results: We have provided a detailed analysis of the biological significance of our findings, linking the identified genes and pathways to the known characteristics of KD. This includes a deeper exploration of the PI3K-Akt signaling pathway and cytokine-cytokine receptor interaction, which are critical for understanding KD’s immune-mediated inflammation (see lines 167 to 178 now).

--Immune Cell Dynamics: We have critically examined the changes in immune cell distribution in KD patients, discussing how these changes correlate with the observed gene expression patterns and their potential impact on disease progression (see lines 179 to 204 now).

--Therapeutic Implications: We have highlighted the potential therapeutic significance of our findings, suggesting that targeting the identified pathways and genes could offer new strategies for managing KD (see lines 206 to 210 now).

**Comment 1:** The rationale for correlating immune cells and gene expression, and how the link between both supports the role of the gene-associated proteins in KD remains unclear.

**Response:**

Thank you for your comment. We have clarified the rationale for correlating immune cells and gene expression in our study:

--Immune Cells in KD: KD involves significant immune cell infiltration (e.g., neutrophils, monocytes) that drives inflammation.

--Gene Expression and Immune Function: The identified DEGs are involved in immune response pathways. Correlating these genes with immune cell levels helps identify potential regulatory relationships.

--Functional Relevance: For example, CCL2, which recruits immune cells, correlates strongly with immune cell abundance, supporting its role in KD inflammation.

--Supporting Protein Roles: The correlation analysis strengthens the evidence that the identified gene-associated proteins (e.g., AURKB, BUB1, TOP2A, CCL2) are functionally relevant to KD pathogenesis.

In summary, the correlation between immune cells and gene expression in our study is based on the well-established involvement of immune cells in KD and the functional relevance of the identified genes in immune response pathways. This link supports the hypothesis that the gene-associated proteins contribute to KD pathogenesis by modulating immune cell behavior and inflammatory responses.

**Comment 2:** The study limitations such as the size of the dataset, the usage of a single KD dataset, the specificity/sensitivity for KD of these potential biomarkers or the lack of experimental validation needs to be discussed.
**Response:**

Thank you for your feedback. We have addressed the study limitations in the manuscript as follows:

--The small sample size and use of a single KD dataset limit the generalizability of our findings and our ability to capture KD patient diversity (see lines 211 to 212 now).

--The study does not show gene expression patterns and immune cell infiltration in different KD subtypes, limiting our understanding of KD pathogenesis (see lines 212 to 213 now).

--We lack experimental validation and have not evaluated the specificity/sensitivity of the identified potential biomarkers for KD (see lines 214 to 215 now).

--We have noted these limitations and emphasized the need for future studies with larger and more diverse datasets, independent validation, and experimental confirmation of our findings (see lines 215 to 216 now).

**Comment 3:** The language needs to be more conservative as there is no proof that the identified hub genes are valid biomarkers, while the study definitely is supporting their role in KD. For example, line 148 “In this study, five hub genes involved in KD (AURKB, BUB1, CCL2, IL-4 and TOP2A) 148 were identified.” could be “In this study, five candidate hub genes for KD (AURKB, BUB1, CCL2, IL-4 and TOP2A) were identified.”.
**Response:**

Thank you for your suggestion regarding the language used in our manuscript. We have carefully revised the text to adopt a more conservative tone, ensuring that our claims are appropriately cautious. For example, we have changed the phrase at line 148 from “hub genes involved in KD” to “candidate hub genes for KD,” clearly indicating that further validation is needed (see lines 161 now). We have also reviewed and adjusted similar statements throughout the manuscript to accurately reflect the preliminary nature of our findings.

**Comment 4:** Similarly, the title needs to be changed as this is far from qualifying as a preclinical study without any experimental data to validate the findings.

**Response:**

Thank you for your insightful feedback. We have revised the title to better reflect the nature of our work as a bioinformatics analysis rather than a preclinical study. The new title is: "Identification of Potential Biomarkers associated with Immune Cell Infiltration Patterns in Kawasaki Disease via Bioinformatics". We believe this title more accurately represents the content and scope of our study..

**Comment 5:** The discussion needs to rely more on previous literature and link the role of identified hub genes with KD as the link remains unclear.

**Response:**

Thank you for your valuable feedback. We have revised the discussion section to incorporate more references to previous literature and to better link the roles of the identified hub genes with Kawasaki Disease (KD). Specifically, we have highlighted how these genes relate to KD pathogenesis based on their known functions and interactions. This should provide a clearer understanding of their potential roles in the disease (see lines 163 to 178 now).

**Comment 6:** In general, the study is not fluid to read, with regular typos/language mistakes making it difficult to read. I would suggest taking advantage of professional language editing.

**Response:**

Thank you for your feedback. In response to your comments, we have carefully reviewed the manuscript and conducted thorough proofreading to correct any typos and language errors. We have also revised the text to enhance clarity and readability. We are committed to ensuring the manuscript is of the highest quality. We apologize for any difficulties caused by language issues and appreciate your suggestion to improve the manuscript.

**Comment 7:** Line 10: There is a missing noun word for the adjective “immune”

**Response:**

We have added the noun "dysregulation" to clarify the term "immune." The phrase now reads: "Inflammation and immune dysregulation play critical roles in Kawasaki disease (KD) pathogenesis (see lines 10 now).

**Comment 8:** Line 12: Please clarify what “immune infiltration situation” refers to.

**Response:**

Thank you for your comment. We have revised "immune infiltration situation" at line 12 to "immune cell infiltration scores" for clearer study objectives (see lines 12 now). We hope this revision provides the necessary clarity.

**Comment 9:** Line 14: Please add more details about GSE73461 such as specifying how many KD and control patients were included, where are they from, when was this set obtained.

**Response:**

We have added more details about the GSE73461 dataset at line 14. The revised sentence now reads: "The GSE73461 dataset, downloaded from the Gene Expression Omnibus (GEO) database, includes 78 KD patients and 55 normal controls collected by Imperial College London from 2015 to 2023, and was analyzed to identify differentially expressed genes (DEGs) (see lines 12 to 15 now)." We hope this provides the necessary information.

**Comment 10:** Line 62: There are two “provide(s)”, please remove one.

**Response:**

Thank you for pointing out the redundancy in the sentence. We have revised the sentence at line 62 to correct the repetition and improve clarity. The updated sentence now reads: "This study aims to identify corresponding biomarkers and immune cell infiltration in KD, thereby providing insight for early diagnosis and targeted therapeutic strategies (see lines 53 to 54 now)." We appreciate your attention to detail.

**Comment 11:** Lines 99-101: The result part would benefit from more description of gene up- and downregulation: in the volcano plot some look significantly more upregulated and downregulated, can you comment on that? |log FC| > 8 or 10.

**Response:**

Thank you for your suggestion regarding the description of gene up- and downregulation in the results section. We have revised lines 99-101 to provide more detailed information about the differentially expressed genes (DEGs) identified in our study. The updated text now reads: "In this study, we identified 1229 DEGs with P-values < 0.05 and |log2 FC|≥ 2, including 720 up-regulated and 509 down-regulated genes. Notably, those with |log2 FC| > 8 are particularly important for understanding specific biological processes or disease states and require further study (Fig. 1) (see lines 95 to 97 now)." We hope this revision provides the additional context and explanation you were looking for.

**Comment 12:** Fig 1B: The heatmap figure does not help the reader to understand the study, the results can be visualized in Fig 1A. I would suggest removing Fig 1B or moving it to the supplementary information.

**Response:**

Thank you for your suggestion regarding Figure 1B. We have carefully considered your feedback and have removed Fig 1B as suggested. The key results are now fully presented in Fig 1A, which provides a clearer and more focused visualization of the data.

**Comment 13:** Lines 103-108: I would suggest commenting on the results, as some of them were to be expected for KD.

**Response:**

Thank you for your suggestion. We have revised lines 103-108 to provide more detailed commentary on the results, highlighting both the expected and novel aspects of our findings. The updated text now reads: "GO and KEGG analysis highlight key biological roles of DEGs. GO analysis shows significant links to acute inflammatory response and plasma membrane components (Fig 2A and S1A Fig), which are expected given KD's characteristic inflammation and vascular involvement. KEGG analysis points to enrichment in pathways like cytokine interactions and PI3K-Akt signaling (Fig 2B and S1B Fig), further supporting KD's known inflammatory and immune-mediated nature. While these findings are consistent with the established pathology of KD, the detailed enrichment patterns offer a more comprehensive view of its molecular mechanisms. Figs 2C and 2D show how DEGs relate to these pathways, emphasizing their importance in our study's biological context and offering new insights into potential therapeutic targets (see lines 99 to 106 now)."

**Comment 14:** Fig. 2: The images are too small, please ensure the text is readable.

**Response:**

Thank you for your comment regarding the readability of the images in Figure 2. We have taken steps to address the issue. Specifically: Figures 2A-2D and 2F (now): We have resized these figures to ensure that the text is clearly readable while maintaining the required dimensions. Figure 2G: We apologize for the inconvenience caused by the size and resolution of Figure 2G. We have encountered difficulties in resizing this particular figure while maintaining the clarity and readability of the text. The current dimensions (8334×9524 pixels) exceed the journal's specified width and height limits (width 789-2250 pixels, height 2625 pixels). We are actively seeking solutions to address this issue and have already reached out to the journal for assistance. Unfortunately, we have not yet received a response. We appreciate your understanding and patience as we work to resolve this technical challenge. We will update Figure 2G as soon as we receive guidance from the journal.

**Comment 15:** Fig. 2A-2F: I would suggest moving some of the figures to a supplementary information document as some of the information is redundant why not uninteresting. This would allow for larger figures and facilitate readability of the manuscript.
**Response:**

Thank you for your suggestion regarding Figure 2. We have taken your feedback into consideration and have moved Fig 2D-2F to Supplementary Fig 1. This change allows us to present the remaining figures in a larger format, enhancing their readability and clarity.

**Comment 16:** Fig 2G: It is impossible to read it due to the size and the identified hubs are then unclear. Please update figure.
**Response:**

Thank you for your comment regarding Figure 2G. We apologize for the difficulty in reading the figure due to its size. We have attempted to resize the figure while maintaining the clarity of the identified hubs, but the current dimensions (8334×9524 pixels) exceed the journal's specified limits (width 789-2250 pixels, height 2625 pixels). We have already contacted the journal for guidance on how to properly format the figure to meet their requirements. Unfortunately, we have not yet received a response. We are actively working on resolving this issue and will update the figure as soon as we receive further instructions from the journal. We appreciate your understanding and patience as we work to address this technical challenge.

**Comment 17:** Fig. 3: I would suggest moving some of the figures (A, B, C, E) to the SI and maybe generating a summary figure to make the results easy to interpret for the reader supporting the statements from line 119 to line 139. Also noting that the immune cell name formatting needs to be corrected.
**Response:**

Thank you for your suggestions regarding Figure 3. We have taken the following actions to address your comments:

--Moved Figures to Supplementary Information: We have moved Fig 3(A, B, C, E) to Supplementary Fig 2 to declutter the main text and focus on the most critical results

--Created a Summary Figure: We have generated a summary figure (Fig S2) that consolidates the key findings and supports the statements (see line 114 to 122). This should make the results easier to interpret for the reader.

--Corrected Immune Cell Name Formatting: We have reviewed and corrected the formatting of immune cell names throughout the manuscript to ensure consistency and clarity.

**Comment 18:** Fig. 3D: The color legend is missing, please add it to the figure.

**Response:**

Thank you for pointing out the missing color legend. Following your suggestion, we have reorganized Figure 3 by moving panels A, B, C, and E to Supplementary Figure 2. As a result, the original Figure 3D is now the new Figure 3A. We have added the color legend to the new Figure 3A to ensure that the data is clearly understandable.

**Reviewer #4:**

**Comment 1:** The paper lacks novelty. There are already published papers related to the same topic of the paper. For example, there is a peer reviewed published paper here https://pubmed.ncbi.nlm.nih.gov/37837870/ named Identification of hub biomarkers and immune-related pathways participating in the progression of Kawasaki disease by integrated bioinformatics analysis. The paper conducted more comprehensive and used more datasets to identify biomarkers for Kawasaki disease.
**Response:**

Thank you for your valuable comments. We have carefully considered your concerns regarding the novelty of our manuscript and would like to provide a clear and concise response.

You have pointed out that our paper may lack novelty due to the existence of a similar study titled "Identification of hub biomarkers and immune-related pathways participating in the progression of Kawasaki disease by integrated bioinformatics analysis". While we acknowledge the similarities in research topics, we believe our study offers distinct and innovative contributions that set it apart from the cited work. Here are the key differences:

--Research Focus: The paper you referred to primarily focuses on identifying biomarkers and immune-related pathways using comprehensive bioinformatics analysis, emphasizing the correlation of these biomarkers with peripheral blood immune cells. Our study specifically explores the association between potential biomarkers and immune cell infiltration patterns in Kawasaki disease, aiming to elucidate how these biomarkers influence disease progression through immune cell interactions.

--Data and Methods: The paper you referred to utilizes three datasets (GSE63881, GSE73463, and GSE73461) and employs methods such as WGCNA, DEGs analysis, PPI network construction, and LASSO regression. Our study primarily conducted an in-depth analysis based on the GSE73461 dataset. For the analysis of immune cell infiltration, we employed the XCell algorithm, a relatively novel method that enables more accurate assessment of the infiltration levels of various immune and non-immune cells. Moreover, our analysis specifically focused on the interactions among immune cells and their correlations with biomarkers, aspects that were not extensively explored in the paper you referred to.

--Results and Conclusions: The paper you referred to identifies key genes (e.g., S100A12, MMP9, TLR2) that are correlated with immune cells. We identify a different set of key genes (e.g., AURKB, BUB1, CCL2,TOP2A) and demonstrate their significant association with specific immune cell types (e.g., monocytes, neutrophils). Our findings highlight the potential for immune cell-targeted therapies, offering new directions for clinical treatment.

--Clinical Implications: The paper you referred to provides a theoretical foundation for biomarker identification in Kawasaki disease. Our study goes further by linking these biomarkers to immune cell infiltration, suggesting practical applications for early diagnosis and targeted treatments.

In summary, while both studies investigate Kawasaki disease, our research offers unique insights into the relationship between biomarkers and immune cell infiltration. We believe our findings significantly contribute to the field and warrant publication.

**Comment 2:** The methodology is too simple, just differential expression analysis, resulting in more than one thousand genes. Additionally, selecting hub genes from the protein-protein interaction (PPI) network is insufficient for establishing reliable biomarkers.
**Response:**

Thank you for your insightful comments regarding the methodology of our study. We understand your concerns about the simplicity of the differential expression analysis and the sufficiency of selecting hub genes from the protein-protein interaction (PPI) network. We would like to provide a detailed response to address these points:

--Differential Expression Analysis: While differential expression analysis is a fundamental step in identifying genes that are significantly altered between conditions, we recognize that it alone is not sufficient to identify reliable biomarkers. In our study, differential expression analysis was only the initial step. We used it to identify a large pool of differentially expressed genes (DEGs) from the GSE73461 dataset, which included 1229 genes with |log2 fold change (FC)| ≥ 2 and an adjusted false discovery rate (FDR) P < 0.05. This step was crucial for narrowing down the candidate genes for further analysis.

--Selection of Hub Genes from the PPI Network: We agree that selecting hub genes solely based on the PPI network is not sufficient. Therefore, we employed a multi-step approach to ensure the reliability of the identified biomarkers: ① Functional and Pathway Enrichment Analysis: After identifying the DEGs, we performed Gene Ontology (GO) and Kyoto Encyclopedia of Genes and Genomes (KEGG) enrichment analyses to understand the biological roles and pathways associated with these genes. This step helped us to identify genes that are involved in critical biological processes and pathways relevant to Kawasaki disease (KD). ② Protein-Protein Interaction (PPI) Network Construction: We constructed a PPI network using the STRING database and identified candidate hub genes based on their degree centrality. This step allowed us to focus on genes that are central to the biological network and likely to play key roles in the disease. ③ Immune Cell Infiltration Analysis: We used the XCell algorithm to evaluate the infiltration levels of 29 immune and non-immune cells. This analysis revealed significant differences in immune cell infiltration between KD patients and normal controls, and we correlated these findings with the identified hub genes. This step provided additional evidence for the involvement of these genes in the immune response associated with KD. ④ Correlation Analysis: We performed Spearman correlation analysis to assess the relationships between the candidate hub genes and the infiltrating immune cells. This analysis further validated the biological relevance of the identified hub genes in the context of KD.

--Integration of Multiple Analytical Approaches:

Our study integrates multiple analytical approaches to ensure the robustness of the identified biomarkers. By combining differential expression analysis, functional enrichment, PPI network analysis, immune cell infiltration analysis, and correlation analysis, we aimed to identify key genes that are not only differentially expressed but also play significant roles in the disease pathogenesis through their interactions with immune cells.

Specifically, we identified five candidate hub genes (AURKB, BUB1, CCL2, IL4, and TOP2A) that showed significant associations with immune cell infiltration and are involved in critical pathways such as PI3K-Akt signaling and cytokine interactions. These genes were further validated through their correlations with specific immune cell types, highlighting their potential as reliable biomarkers for KD. In summary, while differential expression analysis was an essential starting point, our study employed a comprehensive and integrated approach to identify and validate potential biomarkers for Kawasaki disease. We believe that this multi-step methodology provides a more reliable and biologically relevant set of biomarkers compared to using differential expression analysis alone.

**Comment 3:** The paper only used a single microarray dataset, which is outdated and may not reflect current advancements in technology or data availability.
**Response:**

Thank you for your comment regarding the use of a single microarray dataset in our study. We understand your concerns about the potential limitations of using a single dataset and its relevance to current advancements in technology and data availability. Here is our detailed response:

--Choice of Dataset: We acknowledge that using a single dataset may have limitations. However, the GSE73461 dataset was chosen for its comprehensive and well-characterized nature, which includes a substantial number of samples (78 KD patients and 55 normal controls) collected over a recent period (2015–2023). This dataset provides a robust foundation for our initial exploration of potential biomarkers and immune cell infiltration patterns in Kawasaki disease (KD).

--Relevance and Quality: Despite being a single dataset, GSE73461 is one of the most recent and comprehensive microarray datasets available for KD. It offers high-quality data that has been validated through multiple studies, ensuring reliability and reproducibility. The dataset includes detailed clinical annotations, which are crucial for correlating gene expression profiles with disease states and immune cell infiltration patterns.

--Current Advancements and Data Availability: We are aware of the rapid advancements in sequencing technologies and the increasing availability of RNA-seq datasets. However, at the time of our study, comprehensive RNA-seq datasets for KD were limited. The GSE73461 dataset, despite being a microarray dataset, remains one of the most relevant and accessible resources for studying KD. We believe that leveraging this dataset allows us to provide valuable insights into the disease mechanisms and potential biomarkers.

--Future Work: We agree that incorporating additional datasets, particularly RNA-seq data, would enhance the robustness and generalizability of our findings. In future work, we plan to validate our findings using additional datasets, including RNA-seq data, as they become available. This will allow us to further refine our biomarker candidates and explore their potential applications in a broader context.

--Integration of Multiple Datasets: While our current study focuses on a single dataset, we recognize the importance of integrating multiple datasets to capture the full spectrum of KD heterogeneity. We are actively exploring opportunities to collaborate with other research groups to access and analyze additional datasets, which will provide a more comprehensive understanding of the disease.

In summary, while we acknowledge the limitations of using a single microarray dataset, we believe that the GSE73461 dataset provides a valuable and relevant starting point for our research. We are committed to expanding our analysis to include additional datasets in future work to ensure that our findings are robust and reflective of current advancements in technology and data availability.

We believe that these revisions have significantly improved the quality and robustness of our manuscript. We are confident that our study provides valuable insights into the molecular mechanisms underlying Kawasaki disease and offers potential biomarkers for further investigation.

Thank you again for your time and valuable feedback. We look forward to your positive response.

Best regards,

[Rong Wang]
[Department of Pediatrics, Cangzhou People's Hospital, Hebei Province, China]
[Email: [19933278898@163.com](mailto:19933278898@163.com) & [1056980394@qq.com](mailto:1056980394@qq.com)]
[Mar 21 2025]
